# HucMSC exosomes promoted imatinib-induced apoptosis in K562-R cells via a miR-145a-5p/USP6/GLS1 axis

**DOI:** 10.1038/s41419-022-04531-3

**Published:** 2022-01-28

**Authors:** Xiaowen Chen, Yixin Chen, Min Zhang, Hui Cheng, Huirong Mai, Meng Yi, Huanli Xu, Xiuli Yuan, Sixi Liu, Feiqiu Wen

**Affiliations:** 1 Department of Hematology and Oncology, Shenzhen Children’s Hospital, Shenzhen, 518038 China; 2 Shenzhen Institute of Pediatrics, Shenzhen Children’s Hospital, Shenzhen, 518038 China; 3 Department of Oncology, Shenzhen People’s Hospital (The Second Clinical Medical College, Jinan University; The First Affiliated Hospital, Southern University of Science and Technology), Shenzhen, 518020 China; 4 Department of Hematology, Changhai Hospital, Naval Military Medical University, Shanghai, 200433 China

**Keywords:** Cancer therapeutic resistance, Ubiquitylation

## Abstract

Chronic myeloid leukemia (CML) is a myeloproliferative neoplasm with increasing incidence worldwide. Growing evidence suggests that ubiquitin-specific proteases (USPs) play a role in cancer treatment. Dysregulation of miR-146a has been found in both adult and pediatric patients with acute leukemia. Knockdown of glutaminase-1 (GLS1) resulted in inhibition of tumor growth. However, the role of miR-146a-5p/USP6/GLS1 in leukemia and chemoresistance of leukemia cells remains to be elucidated. In the current study, USP6 level was increased in bone marrow aspiration specimens of patients with CML and associated with poor prognosis. USP6 was significantly upregulated in imatinib (IM)-resistant clinical samples compared with IM-sensitive samples. USP6 overexpression significantly inhibited IM-induced apoptosis of leukemia cells. Overexpressing USP6 significantly increased GLS1 ubiquitination to decrease GLS protein. A mechanism study indicated that USP6 regulation of IM resistance of CML cells was GLS1 dependent and regulated by miR-146a-5p. Administration of human umbilical cord mesenchymal stem cell (hucMSC) exosomes promoted IM-induced cell apoptosis through miR-145a-5p/USP6. Therefore, hucMSC exosomes promoted IM-induced apoptosis of K562-R cells by suppressing GLS1 ubiquitination to increase GLS protein via miR-146a-5p and its target GLS1. The findings highlight the importance of miR-146a-5p/USP6/GLS1 signaling in chemoresistance of leukemia and provide new insights into therapeutic strategies for chemoresistant leukemia.

## Introduction

Chronic myeloid leukemia (CML) is a myeloproliferative neoplasm with uncontrolled proliferation of granulocytes [1]. It accounts for approximately 15% of newly diagnosed adult leukemia [1, 2]. The incidence cases, death cases, and disability-adjusted life years of CML in countries with a lower socio-demographic index are increasing [3]. CML had been traditionally treated with conventional chemotherapy with hydroxyurea, busulfan, or imatinib (IM), a tyrosine kinase inhibitor [3, 4]. Although the outcomes of CML have improved dramatically, survival rates are unsatisfactory [5].

Protein ubiquitination is implicated in almost all aspects of biological processes [6]. It is a multistep enzymatic process that attaches ubiquitin to a substrate and directs the substrate protein for proteolytic degradation mainly via the ubiquitin–proteasome system (UPS) [7]. Ubiquitin-specific proteases (USPs) are one of the five identified classes of deubiquitinases, which play a very important role in deubiquitination and ubiquitin recycling [8]. Dysfunction of the ubiquitin system leads to the development of various human diseases, including cancer [9]. USP-specific inhibitors have been used as antiviral and anticancer agents. For example, a small molecule, ML364, has been shown to inhibit USP2 for cell cycle arrest [10]. Yao et al. have shown that USP7 silencing increased the sensitivity of multiple myeloma cells to bortezomib [11].

Exosomes are small extracellular vesicles (30–150 nm) that play a role in cell–cell communication [12]. Exosomes from different origins have different types of mRNAs or miRNAs [13]. Subsequently, exosomal miRNAs or mRNAs can be delivered to target cells [14]. More than 150 miRNAs have been found in MSC-derived exosomes, and many miRNAs play a role in pathological processes [15, 16]. Human umbilical cord mesenchymal stem cells (hucMSCs) have been useful in the treatment of several diseases by secreting paracrine factors, including exosomes [17]. It has been reported that exosomes from hucMSCs contain higher miR-146a-5p levels [18, 19]. HucMSC-derived exosomal miR-146a-5p reduces microglial-mediated neuroinflammation after ischemic stroke [20] and increases the sensitivity of ovarian cancer cells to docetaxel and taxane [21]. Dysregulation of miR-146a-5p has been found in both adult and pediatric patients with acute leukemia [22].

Glutaminase-1 (GLS1), a mitochondrial enzyme that is expressed in most tissues, metabolizes glutamine to glutamate and ammonia to promote cancer cell proliferation primarily through the formation of tricarboxylic acid cycle intermediates [23, 24]. Increasing evidence suggests that glutamine metabolism by GLS1 plays an important role in different types of cancers [25]. Moreover, a growing body of evidence supports that GLS1 is involved in chemoresistance. For instance, Masamha et al. have shown that targeting GLS1 by siRNA efficiently sensitized ovarian cancer cells to cisplatin treatment [26]. Another study by Fu et al. demonstrated that knockdown of GLS1 using siRNA resensitized resistant breast cancer cells to Taxol [27].

However, the roles of dysregulation of protein ubiquitination, hucMSC exosomal miR-146a-5p, and GLS1 in CML remain to be elucidated. The present study aimed to investigate the role of miR-146a-5p/USP6/GLS1 chemoresistance of leukemia cells and decipher the molecular pathways that were necessary for such effects.

## Materials and methods

### Clinical samples

The study complied with the principles of the 1975 Declaration of Helsinki and obtained approval from the Institutional Ethical Review Committee of Changhai Hospital, Naval Military Medical University (CHEC2021-002). Written informed consents were obtained from the participants. The clinical samples were collected from Changhai Hospital (Cohort 1 and Cohort 2). Cohort 1 consisted of 20 cases of IM-sensitive and 20 cases of IM-resistant (relapsed) bone marrow aspiration specimens collected through retrospective analysis for quantitative polymerase chain reaction (qPCR) analysis. Three samples were collected from each group for Western blot analysis. Fifteen normal bone marrow puncture specimens served as controls. Cohort 2 consisted of 85 CML bone marrow specimens for detecting USP6 expression and analysis of the correlation of USP6 and prognosis.

### Cell culture

K562 cells were provided by Shanghai Cell Bank (Shanghai, China) and authenticated by STR before shipping. Mycoplasma contamination was tested if concerned. Cells were cultured in gradually increased IM concentrations over a period of several months to collect K562-R cells. All cells were cultured in DMEM with 10% FCS at 37 °C in a 5% CO_2_-humidified atmosphere.

### Quantitative PCR (qPCR)

Total RNA was extracted with TRIzol (Beyotime, Shanghai), reverse-transcripted into cDNA with a commercial kit (#K1622, Thermo). Then, gene or miRNA expression was analyzed using qPCR with U6/GAPDH as a control and using primers (Table S1). The 2 ^−ΔΔCt^ formula was used to quantify gene expression.

### Protein isolation and Western blot analysis

Cells were lysed in RIPA buffer (Beyotime, Suzhou). Moreover, 20 μg of protein extracts were resolved on a 10% or 15% SDS polyacrylamide gel and electroblotted onto a nitrocellular membrane (Millipore). After blocking, membranes were probed with first antibodies (Table S2) and second antibody. Immunoreactivity signals were visualized using ECL chromogenic substrate (Bio-Rad Laboratories).

### Construction of plasmids and preparation of lentivirus

Short-hairpin RNAs (shRNAs) (Table S3) were ligated into linearized pLKO.1 (OriGene, Rockville, MD). cDNAs of USP6 or GLS1 were ligated into pLVX-Puro (OriGene). Viruses were packaged in 293 T cells with psPAX2 and pMD2G.

### Apoptosis assay

Cells were treated as indicated. After treatment, cells were washed and stained with Annexin V and PI (Beyotime) and subjected to flow cytometry analysis (BD, Shanghai).

### Immunoprecipitation (IP) assays

Proteins were incubated with anti-USP6 (Bethy Laboratories, A305-225), anti-GLS1 (Cell Signaling Technology, #56750), or control IgG (Beyotime) for 1 h, followed by incubation with protein A/G-agarose for 3 h at 4 °C. Precipitates were washed and submitted for Western blot analysis.

### Glutamine assay

The glutamine concentrations were measured using a glutamine assay kit (Abcam). Briefly, cells (2 × 10^6^) were washed twice with cold phosphate-buffered saline, resuspended in ice-cold hydrolysis buffer, homogenized, and centrifuged at 4 °C at 10,000 × *g* for 10 min to collect the supernatant. Ice-cold perchloric acid (PCA) was used for deproteinization and KOH (2 M) was used to precipitate excess PCA. Then, samples were incubated with glutamine-reaction mix at 37 °C for 60 min, and absorbance at 450 nm was recorded on a microplate reader.

### miRNA interference and overexpression

Cells were seeded in six-well plates (2 × 10^5^ cells/well), cultured overnight, and transfected with the miR-146a-5p mimic (50 nM, 5′-UGAGAACUGAAUUCCAUGGGUU-3′), inhibitor (50 nM, 5′-AACCCAUGGAAUUCAGUUCUCA-3′), or negative control RNA (50 nM, 5′-CAGUACUUUUGUGUAGUACAA-3′) using Lipofectamine 2000 (Invitrogen).

### Luciferase assay

Binding sites of miR-146a-5p in the 3′-UTR of USP6 were predicted. Wild-type (WT) and mutant (Mut) sequences were ligated to pGL3 vector, which was transfected to 293 T cells with miR-146a-5p mimics/inhibitor using Lipofectamine 2000. Luciferase activity was measured after 2 days using a commercial kit (Beyotime, Beijing).

### Exosome uptake assay

HucMSC exosomes were stained with PKH26 Dye (Sigma-Aldrich) to determine their internalization by K562-R. Exosomes were labeled with PKH26 at ambient temperature (20–25 °C) for 5 min and incubated with K562-R cells for 24 h at 37 °C. K562-R cells were washed twice and fixed in 4% paraformaldehyde. DAPI was used to stain nuclei.

### Animal study

Animal experiments were approved by the Animal Care Committee of Shenzhen Children’s Hospital (SUMC2017-086). NOD-SCID mice (female, 6–8 weeks) were purchased from SLRC Laboratory Animal (Shanghai). K562-R cells were administered to the mice through the tail vein (2 × 10^6^/mouse). Mice were randomized into three groups (n = 10 in each group): (1) untreated group, (2) IM-treated group (150 mg/kg/day), and (3) IM (150 mg/kg/day) and hucMSC-exo-treated (5 μg/day) group. Mice that did not receive any treatment were used as controls (*n* = 10). Mice were sacrificed on day 90, and the blood and spleen were collected.

### Statistical analysis

All experiments were performed at least three times. Statistical analysis was conducted using GraphPad Prism 8.4.2 (La Jolla, CA). Whether the data were normally distributed was tested using the one-sample Kolmogorov–Smirnov test. The measurement data between two groups were compared using the Student’s t-test if they were normally distributed and the variation was comparable. One-way analysis of variance test was performed among three or more groups if the variation were comparable. When the data were shown, the skewed distribution, comparisons were performed by nonparametric tests. *P*-values <0.05 were considered significant.

## Results

### USP6 was highly expressed in bone marrow aspiration specimens of patients with CML and associated with poor prognosis

**Fig. 1:**
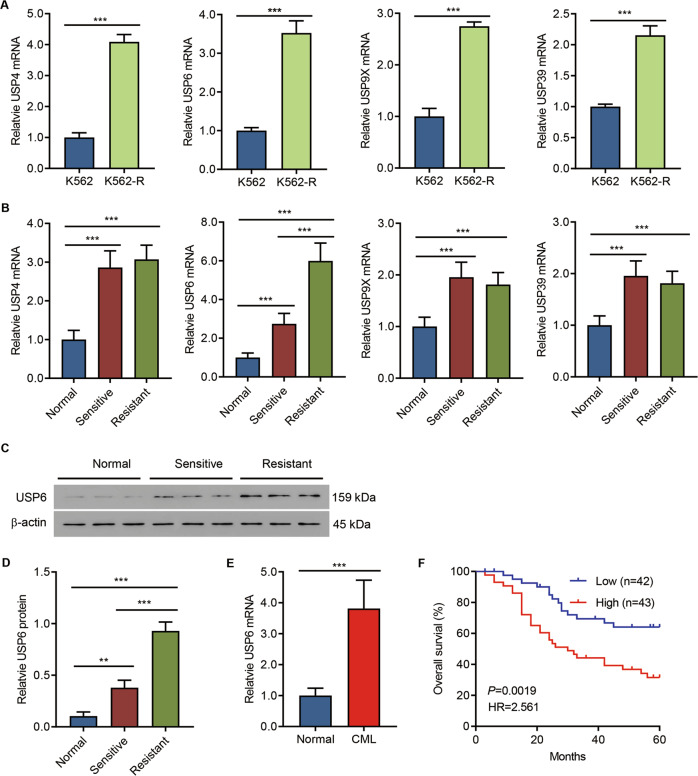
USP6 was highly expressed in bone marrow aspiration specimens of patients with CML and associated with poor prognosis. **A** mRNA levels of USP family members in IM-resistant strain K562-R and its parent cell line K562 were detected by qPCR. **B** qPCR detection of mRNA levels of USP4, USP6, USP9x, and USP39 in clinical samples (bone marrow aspiration specimens from 15 normal controls, 20 patients with IM-sensitive CML, and 20 patients with IM-resistant CML, Cohort 1). **C**, **D** Western blot detection of levels of USP6 in nine clinical samples from Cohort 1 (three from each group). **E** The mRNA level of USP6 in clinical samples (bone marrow specimens from 15 normal controls and 85 patients with CML, Cohort 2). **F** Survival analysis was performed between patients with CML with low-USP6 or high-USP6 expression in Cohort 2 (log-rank test). **P < 0.01, ***P < 0.001.

To analyze the clinical relevance of USPs in CML, we first generated IM-resistant K562-R cell lines using the parental K562 cells. The IC50 (half-maximal inhibitory concentration) of IM was assessed, and IM-resistant cells showed a significant higher IC50 than IM-sensitive cells (Fig. S1). Then, the mRNA levels of USP family members in IM-resistant or IM-sensitive cells were measured. Data indicated that USP4, USP6, USP9x, and USP39 levels were significantly increased in IM-resistant cells (Fig. 1A and Fig S2). qPCR analysis was conducted to further analyze the USP4, USP6, USP9x, and USP39 expression in the clinical samples of Cohort 1, and the results suggested that, compared with IM-sensitive samples, only USP6 was significantly upregulated in IM-resistant samples (Fig. 1B), which was confirmed by Western blot analysis (Fig. 1C, D). Therefore, USP6 was selected for the analysis in this study. The USP6 mRNA level was further measured in the samples of Cohort 2, and the qPCR results demonstrated that USP6 was significantly upregulated in patients with CML compared with that of normal controls (Fig. 1E). Furthermore, our results also suggested that a higher USP6 level correlated with poorer survival (Fig. 1F). The data suggested that USP6 was involved in chemoresistance.

### USP6 regulated the IM resistance of K562 cells

**Fig. 2:**
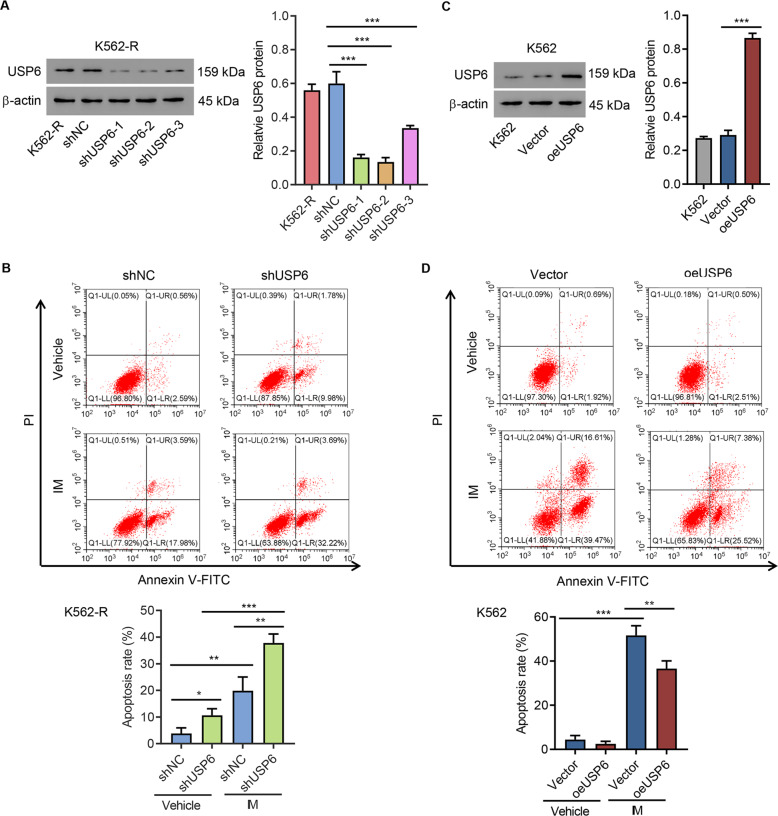
USP6 regulated IM-induced apoptosis. **A** K562-R was infected with USP6 gene-interference lentivirus (shUSP6-1, -2, and -3) or control lentivirus (shNC), Western blot analysis was used to detect USP6 expression. **B** K562-R was infected with shUSP6-1 or shNC for 24 h, treated with 1 μM IM or vehicle (DMSO) for 48 h, and stained with Annexin V/PI, and flow cytometry was used to measure apoptosis. **C** K562 cells were infected with USP6 gene overexpression lentivirus (oeUSP6) or control lentivirus (Vector), and the expression of USP6 was detected by Western blot analysis. **D** At 24 h after K562 cells were infected with oeUSP6 or Vector, they were treated with IM or DMSO for 48 h, stained, and used to detect apoptosis. *P < 0.05, **P < 0.01, ***P < 0.001.

To confirm the involvement of USP6 in IM resistance, USP6 was silenced in K562-R cells using lentivirus carrying either shUSP6 or shNC. Western blot analysis confirmed that USP6 was successfully knocked down (Fig. 2A). USP6-silencing K562-R cells or shNC-K562-R cells were treated with IM (1 μM) or DMSO for 48 h, stained by Annexin V/PI, and subjected to flow cytometry analysis. The results suggested that silencing of USP6 significantly increased IM-induced apoptosis (Fig. 2B). Next, USP6 was overexpressed in K562 cells using lentivirus carrying either USP6 cDNA or empty vector. Western blot analysis confirmed that USP6 was successfully overexpressed (Fig. 2C). Then, USP6-overexpressing K562 or control cells were treated with IM (1 μM) for 48 h and subjected to apoptosis assay. The results suggested that USP6 overexpression significantly inhibited IM-induced apoptosis (Fig. 2D). These findings suggested that USP6 regulated the IM resistance of K562 cells.

### USP6 interacted with GLS1 and inhibited GLS1 ubiquitination in CML cells

**Fig. 3:**
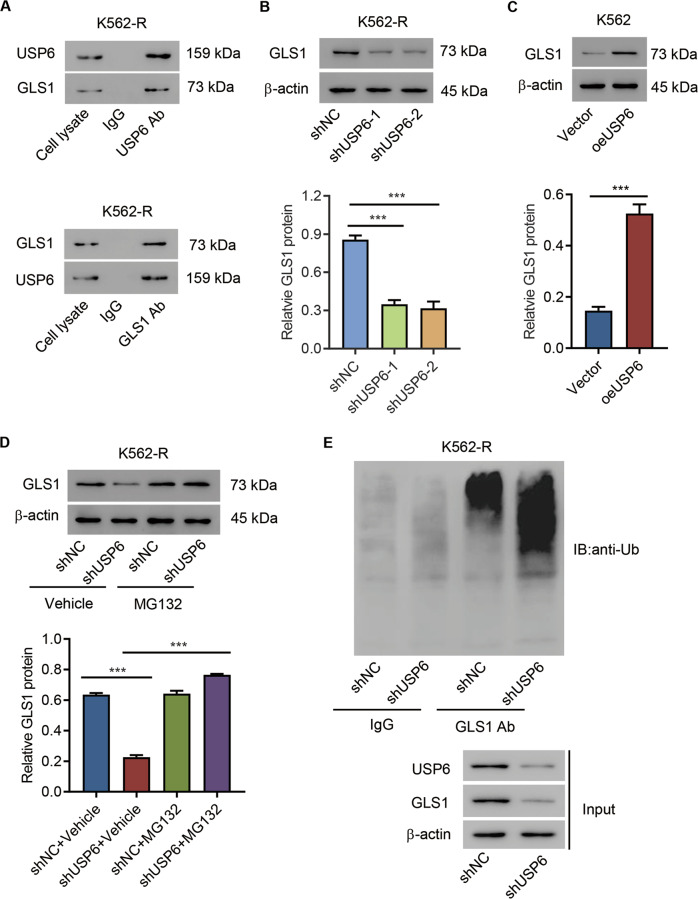
USP6 interacted with GLS1 and inhibited GLS1 ubiquitination in CML cells. **A** IP analysis of the interaction between GLS1 and USP6 in K562-R cells. **B** Western blot analysis of GLS1 protein levels in USP6-silencing K562-R cells. **C** Western blot analysis of GLS1 protein levels in USP6-ovexpressing K562 cells. **D** Western blot analysis of GLS1 expression in USP6-silencing K562-R cells treated with 10 μM MG132 or vehicle (DMSO) for 4 h. **E** IP analysis of the ubiquitination of GLS1in USP6-silencing K562-R cells. ***P < 0.001.

To elucidate how USP6 was involved in IM resistance, we first determined USP6-binding proteins using IP-proteomic analysis and found that GLS1 was one of the top-ranked proteins (Table S4 and Fig S3). Published data have demonstrated that GLS1 is closely related to cancer and drug resistance [27], so we focused on GLS1 in this study. We first performed a Co-IP assay using either anti-USP6 antibody or anti-GLS1 antibody to confirm the potential interaction between USP6 and GLS1 in IM-resistant cells (Fig. 3A). Then, GLS1 expression in USP6-silencing K562-R cells or USP6-overexpressing K562 cells was measured by both qPCR and Western blot analysis. The results suggested that, at the protein level, USP6 silencing significantly suppressed GLS1 expression, while overexpressing USP6 significantly increased GLS1 expression (Fig. 3B, C). However, neither silencing USP6 nor overexpressing USP6 affected GLS1 expression at the mRNA level (data not shown). Next, the effect of a proteasome inhibitor (MG132, 10 μM) on GLS1 expression in USP6-silencing K562-R cells was evaluated. Western blot results suggested that proteasome-inhibitor treatment inhibited GLS1 protein downregulation caused by USP6 silencing (Fig. 3D). IP assay results demonstrated that USP6 silencing promoted GLS1 ubiquitination (Fig. 3E). Together, these findings indicated that USP6 interacted with GLS1 and inhibited GLS1 ubiquitination.

### GLS1 mediated the effects of USP6 in IM resistance of CML cells

**Fig. 4:**
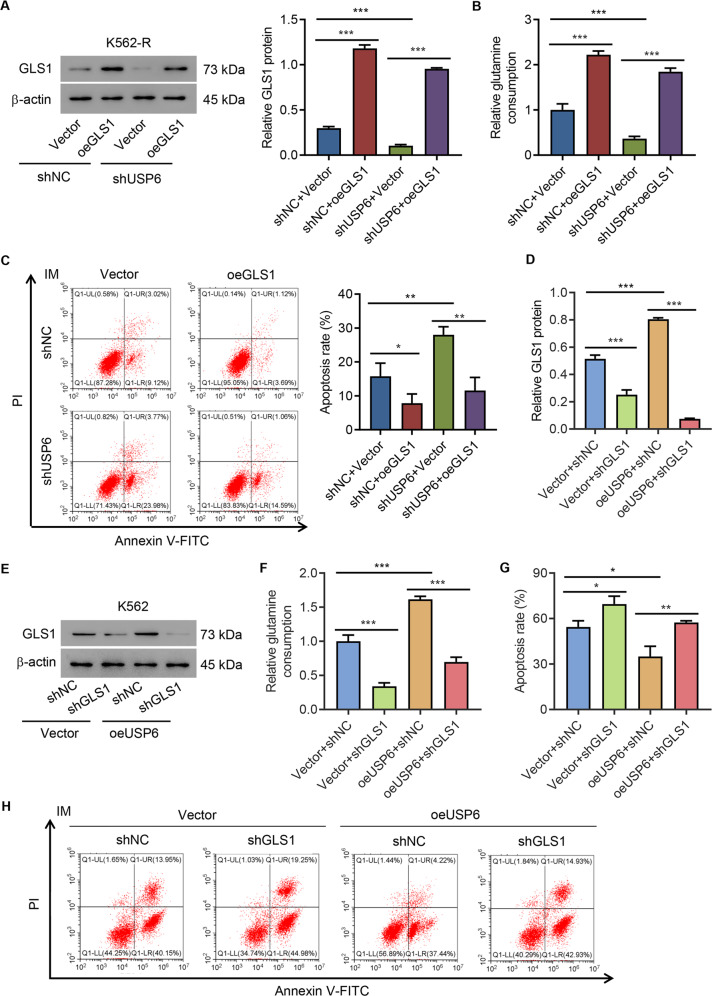
GLS1 mediated the effects of USP6 in IM resistance of CML cells. **A**–**C** K562-R cells were infected with shUSP6-1 or shNC and overexpressed GLS1 (oeGLS1). **A** Western blot analysis of GLS1 expression. **B** Biochemical detection of glutamine uptake. **C** Flow cytometric detection of apoptosis treated by 1 μM IM or DMSO. K562 cells were infected with USP6 gene overexpression lentivirus (oeUSP6) or empty vector (Vector) and interfered with GLS1 (shGLS1). **D**, **E** Western blot analysis of GLS1 expression. **F** Biochemical detection of glutamine uptake. **G**, **H** Flow cytometric detection of apoptosis. *P < 0.05, **P < 0.01, ***P < 0.001.

To study the role of GLS1 in IM resistance of CML cells, USP6 was silenced in K562-R cells using lentivirus carrying shUSP6-1 or shNC. Then, GLS1 was overexpressed (oeGLS1) in USP6-silencing K562-R cells that were confirmed by Western blot analysis (Fig S4A and Fig. 4A). Glutamine uptake by the above-mentioned cell lines was measured by biochemical methods. The results indicated that GLS1 overexpression diminished USP6-knockdown-induced decrease in glutamine uptake in K562-R cells (Fig. 4B). Data also supported that GLS1 overexpression ameliorated USP6-knockdown-induced promotion of IM-induced apoptosis (Fig. 4C). Next, USP6 was overexpressed in K562 cells using lentivirus carrying USP6 cDNA or empty vector. Then, GLS1 was silenced in the USP6-overexpressing K562 cells that were confirmed by Western blot analysis (Fig S4B and Fig. 4D, E). GLS1 knockdown diminished USP6-overexpression-induced increase in glutamine uptake in K562 cells (Fig. 4G) and ameliorated USP6-overexpression-induced inhibition of IM-induced apoptosis (Fig. 4G, H). The results indicated that USP6 regulation on IM resistance of CML cells was GLS1 dependent.

### USP6 was a target of miR-146a-5p

**Fig. 5:**
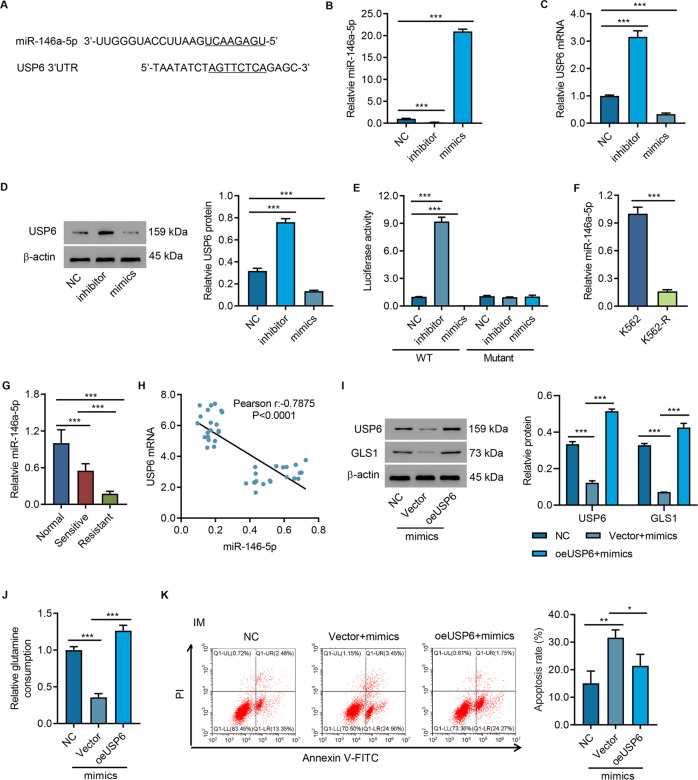
USP6 was a target of miR-146a-5p. **A** Bioinformatic analysis of miR-146a-5p-binding sites in 3′-UTR of USP6. The underlined nucleotides are the complementary sequences to miR-146a-5p seed sequences. **B**, **C** 293 T cells were transfected with miR-146a-5p mimics, inhibitors, or controls (NC), and miR-146a-5p levels, (**B**) USP6 mRNA, and protein levels (**C**, **D**) were analyzed. **E** Luciferase reporter activity in 293T cells transfected with miR-146a-5p mimics, inhibitors, or NC and wild-type USP6 (WT-USP6) or mutant USP6. **F** qPCR analysis of miR-146a-5p levels in K562-R and K562. **G** qPCR analysis of miR-146a-5p levels in clinical samples (bone marrow aspiration specimens from 15 normal controls, 20 patients with IM-sensitive CML, and 20 patients with IM-resistant CML, Cohort 1). **H** Correlation analysis of USP6 and miR-146a-5p in bone marrow aspiration specimen from 40 patients with CML. **I** Western blot analysis of USP6 and GLS1 in K562-R cells overexpressed with miR-146a-5p mimics and USP6. **J** Biochemical detection of glutamine uptake. **K** K562-R cells were overexpressed with miR-146a-5p mimics and USP6 for 24 h and then treated with IM or DMSO for 48 h and used for flow cytometry. *P < 0.05, **P < 0.01, ***P < 0.001.

To study how USP6 was regulated in CML, we conducted literature search and found that miR-146a-5p was downregulated in patients with IM-resistant CML and involved in signaling the presence of DNA damage and activating cell cycle checkpoint [28]. Then, we performed a bioinformatic analysis of USP6, and the results indicated a potential binding site of miR-146a-5p in the 3′UTR of USP6 (Fig. 5A). Next, miR-146a-5p mimics, inhibitor, or control (NC) was transfected into 293T cells (Fig. 5B). miR-146a-5p mimics caused upregulation of USP6, while miR-146a-5p inhibitors decreased USP6 at the protein level. In contrast, miR-146a-5p mimics decreased USP6, while miR-146a-5p inhibitors increased USP6 at mRNA and protein levels (Fig. 5C, D). USP6 3′UTR-WT/3′UTR-Mut and miR-146a-5p inhibitor or mimic was cotransfected into 293T cells. Luciferase assay showed that miR-146a-5p mimics significantly increased the activity of USP6 3′UTR-WT, which was significantly inhibited by miR-146a-5p inhibitor. Mutation of USP6’s miR-146a-5p-binding site blocked miR-146a-5p’s effect on USP6 3′UTR (Fig. 5E). The data suggested that miR-145-5p inhibited USP6 via binding to its 3′UTR. Then, we checked the miR-146a-5p levels in IM-resistant or IM-sensitive cells and clinical samples from Cohort 1. qPCR results indicated that miR-146a-5p was significantly decreased in IM-resistant cells (Fig. 5F). Compared with normal controls, miR-146a-5p was sharply decreased in IM-sensitive patients and further decreased in IM-resistant patients (Fig. 5G). Pearson’s correlation analysis showed that miR-146a-5p was negatively correlated with USP6 (Fig. 5H).

Next, miR-146a-5p mimics were transfected into cells, and USP6 was overexpressed (Fig. 5I). Transfection of miR-146a-5p mimics resulted in a significant decrease in glutamine uptake (Fig. 5J) and significantly promoted IM-induced cell apoptosis (Fig. 5K), which were all abolished by USP6 overexpression (Fig. 5I–K). These findings indicated that miR-146a-5p targeted USP6.

### HucMSC exosome promoted apoptosis of K562-R cells through miR-145a-5p-USP6

**Fig. 6:**
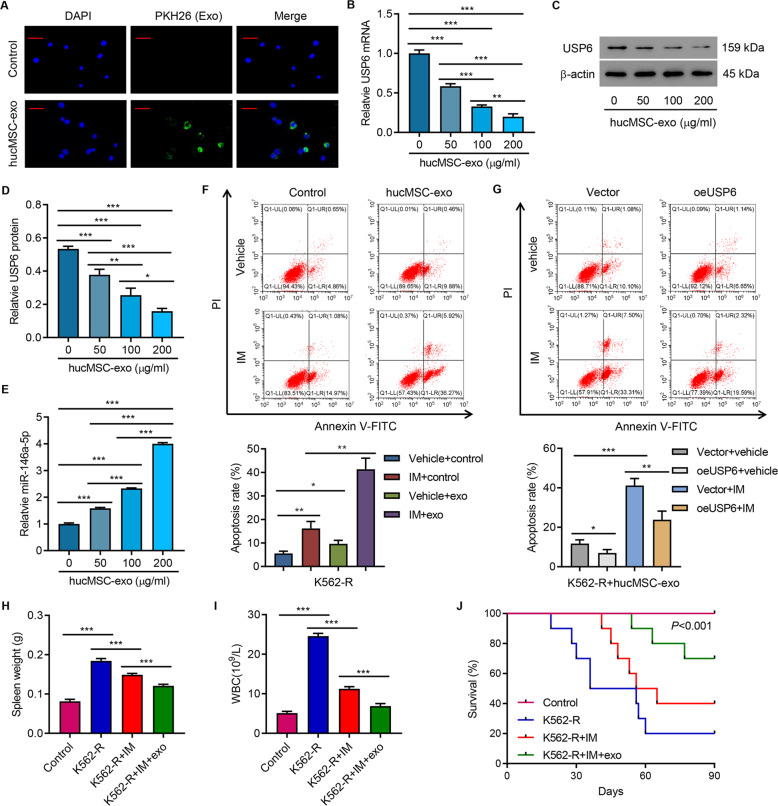
HucMSC exosome promoted IM-induced apoptosis in K562-R cells. **A** PKH26-labeled hucMSC-exo was uptaken by K562-R cells. Scale bar: 50 μm. **B**–**E** The expression of USP6 and miR-146a-5p in K562-R cells treated by different hucMSC-exo concentrations (0, 50, 100, and 200 μg/mL) for 24 h. **F** Flow cytometry analysis of apoptosis of K562-R cells treated by hucMSC-exo (100 μg/mL) alone or in combination with 1 μM IM for 48 h. **G** Flow cytometry analysis of apoptosis of USP6-overexpressing K562-R cells treated by hucMSC-exo (100 μg/mL) alone or in combination with 1 μM IM for 48 h. In vivo tumor formation assays. K562-R was administered to each mouse. On day 90, mice were sacrificed and (**H**) spleen weight, (**I**) white-blood cell count, and (**J**) survival curve were analyzed. *P < 0.05, **P < 0.01, ***P < 0.001.

Next, this study aimed to examine the effect of hucMSC exosomal miR-145a-5p on CML. HucMSC exosomes were first isolated and characterized (Fig S5). Coculture assay indicated that hucMSC exosomes could be uptaken by K562-R cells (Fig. 6A). Administration of hucMSC exosomes decreased USP6 expression and increased miR-146a-5p in a concentration-dependent manner (Fig. 6B–E) and promoted IM-induced cell apoptosis (Fig. 6F). Overexpression of USP6 abolished IM-promoted apoptosis of K562-R cells (Fig. 6G). Then, hucMSCs were transfected with miR-146a-5p mimics, inhibitor, or control (NC). Transfection of miR-146a-5p mimics significantly increased the miR-146a-5p level in both hucMSCs and hucMSC exosomes, while transfection of miR-146a-5p inhibitor significantly decreased the miR-146a-5p level in both hucMSCs and hucMSC exosomes (Fig S6A). Treatment of K562-R cells with inhibitor exo increased USP6 expression and GLS1 at the protein level (Fig S6B), increased glutamine uptake (Fig S6C), and decreased IM-induced apoptosis (Fig S6D), while treatment of K562-R cells with mimic exo significantly decreased the expression of USP6 and GLS1 at the protein level (Fig S6B), decreased glutamine uptake (Fig S6C), and increased IM-induced apoptosis (Fig S6D).

K562-R cells were administered to mice to further study the role of hucMSC-exo in IM resistance. Compared with the nontreatment group, IM treatment significantly decreased the spleen weight (Fig. 6H) and white-blood cell count (Fig. 6I), both of which were further decreased by the administration of hucMSC exosomes (Fig. 6H and I). Consequently, IM treatment significantly increased survival rate, which was further increased by the administration of hucMSC exosomes (Fig. 6J). These data suggested that hucMSC exosomes promoted IM-induced apoptosis of K562-R cells through miR-145a-5p/USP6.

## Discussion

This study first reported that USP6 was significantly increased in bone marrow aspiration specimens of patients with CML, which was significantly associated with poor prognosis. Further studies showed that USP6 interacted with GLS1 and inhibited GLS1 ubiquitination in CML cells. Overexpression of USP6 significantly inhibited IM-induced apoptosis via regulating GLS1. The mechanism study indicated that the effects of USP6/GLS1 were regulated by miR-146a-5p in hucMSC exosomes. For the first time, our study indicated that hucMSC exosomes promoted apoptosis of K562-R cells via a miR-146a-5p/USP6/GLS1 axis.

As a ubiquitin-specific protease, USP6 is overexpressed in benign tumors, such as nodular fasciitis and aneurysmal bone cyst [29]. USP6 has also been shown to confer sensitivity to interferon-mediated apoptosis in Ewing sarcoma [30]. In the current study, we showed that miR-146-5p bound to the 3′UTR region of USP6 gene that negatively regulates USP6 expression. This was further supported by the findings that overexpressing USP6 abolished the effects of miR-146a-5p overexpression. These results not only increase our knowledge of miR-146a-5p/USP6 signaling but also broaden our understanding of chemoresistance in CML cells.

Cancer cells undergo a reprogrammed metabolism to maintain bioenergetics and biosynthesis [31]. Cancer cells adapt to metabolic reprogramming through adjusting the uptake and catabolism of nutrients [32]. Glutamine, one of the major nutrients involved in various aspects of cancer metabolism, serves not only biosynthesis but also tricarboxylic acid cycle [33, 34]. Jiang et al. have reported that glutamine is required by the transition from monolayer culture to anchorage-independent culture [35]. Demas et al. have indicated that glutamine metabolism drives the growth of breast cancer [36]. Converting glutamine to glutamate, GLS1 has attracted increasing attention in cancer and cancer treatment. For instance, GLS1 inhibition suppressed tumor growth and metastasis [24]. Similarly, regulating the expression levels of GLS1 via ubiquitination or deubiquitination is also critical for cancer progression and cancer treatment. For example, Zhao et al. have shown that inhibiting glutamine metabolism caused apoptosis of cancer cells via regulation of GLS1 ubiquitination [37]. Studies also indicate that inhibiting GLS ubiquitination promoted glutaminolysis [38]. GLS1 has also been implicated in chemoresistance. For example, the expression of GLS1 has been increased in Taxol-resistant cells and silencing of GLS1 resensitized cells [27]. A previous investigation reported that GLS1 was upregulated in metastatic ovarian cancer and silencing GLS1 sensitized cancer cells to cisplatin [39]. In the current study, we proved that USP6 inhibited GLS1 ubiquitination to increase GLS1 protein level. This demonstrated that overexpressing USP6 suppressed GLS1 ubiquitination or degradation, resulting in promotion of glutaminolysis and inhibition of apoptosis of CML cells.

Chemotherapeutics can decrease tumor volume or induce short-term remission [40]. However, cancer cells obtained resistance to chemotherapy over time. Drug resistance remains a hurdle to achieve cure in patients with cancer [41]. MSC-derived exosomes can be modified and applied in cancer treatment and promotion of chemosensitivity of cancer cells [42]. For example, miR-302a from hucMSC exosomes suppressed cancer cell growth and migration [42]. In another study, hucMSC-derived exosomes sensitized K562 cells to IM [43]. Here, the downregulation of miR-146a-5p in patients with CML, especially in patients with IM resistance, suggests an additional role in conferring IM resistance. CML is a myeloid neoplasm caused by the BCR-ABL fusion gene that causes dysregulated cellular proliferation and apoptosis resistance via interference in downstream signaling pathways. Recently, it is reported that the miR-150 levels were negatively correlated with *BCR-ABL* transcript level and significantly upregulated following reduction of BCR-ABL tyrosine kinase activity [44]. The expression levels of miR-31, miR-155, and miR-564 were also decreased in CML and influenced by BCR-ABL activity [45]. Thus, future studies should be conducted to validate the regulation of miR-146a-5p by BCR-ABL. The putative gene targets of miR-146a-5p, ATRIP, and ATR encode for two mutually dependent kinases that are essential for signaling the presence of DNA damage and activating cell cycle checkpoint [28]. IM resistance due to point mutations in the BCR-ABL kinase domain was almost immediately identified in the trial phases of the drug [46]. Here, we discovered an extremely important role of hucMSC exosomal miR-145a-5p in regulating chemosensitivity of leukemia cells. Moreover, glutaminase inhibitors sensitize CML cells to IM treatment [47]. Similarly, miR-145a-5p targeted USP6 to regulate GLS1 ubiquitination and degradation, which eventually promoted IM-induced apoptosis in K562-R cells. Lysosomal dysfunction has been shown to have a profound impact on cancer cell growth and survival [48], suggesting that the lysosome is an attractive therapeutic target in cancer therapeutics. Apart from the GSL1, the other protein identified in IP data is a lysosomal serine protease (TPP1), suggesting that USP6 overexpression can influence degradation via proteosome and lysosomes. Stabilizing YB-1, a well-known multifunctional transcription factor and an oncoprotein in cancers, also contributes to USP47-mediated IM resistance in CML [49]. USP15-induced deubiquitination of caspase-6 promotes CML cell apoptosis and inhibits IM resistance [50]. Targeting the USP10/SKP2 axis is a potential strategy to overcome IM resistance in patients with CML [51]. These results broaden our knowledge of chemoresistance in CML, and the role of other substrates mediated by USP6 in regulating IM resistance in CML should be further conducted. Other limitations of the study are as follows: only one cell line (K562/K562-R) was used, and future studies using other types of CML cell lines should be conducted to validate the findings of this study. To further elucidate the role of miR-145a-5p/USP6/GLS1 in chemoresistance of CML, an orthotopic mouse model might be useful.

In conclusion, hucMSC exosomes promote IM-induced apoptosis in K562-R cells by suppressing GLS1 ubiquitination to increase GLS protein via miR-146a-5p and its target USP6. The findings highlight the importance of miR-146a-5p/USP6/GLS1 signaling in chemoresistance of CML and provide new insights into therapeutic strategies for chemoresistant CML.

## Supplementary information

supplemental

aj-checklist

CERTIFICATE OF EDITING

co-authors’ email responses

## Data Availability

All data generated or analyzed during this study are included in this published article (and its supplementary information files).
