# Supplementary material for: HucMSC exosomes promoted imatinib-induced apoptosis in K562-R cells via a miR-145a-5p/USP6/GLS1 axis
Source: Cell Death Dis. 2022 Jan 28;13(1):92. doi: 10.1038/s41419-022-04531-3 (PMC8799639; doi:10.1038/s41419-022-04531-3)
Supplement: Supplementary file 1 — supplemental [file 41419_2022_4531_MOESM1_ESM.docx]

**Table S1.** Primer sequence.

| Homo sapiens ubiquitin specific peptidase 1 (USP1), transcript variant 2, mRNA NM_001017415.1  Primer F 5' GCTAAATCCTTGGGCTATG 3'  Primer R 5' CTTGTGTAGAAGCGAACTG 3' |
| --- |
| Homo sapiens ubiquitin specific peptidase 2 (USP2), transcript variant 3, mRNA NM_001243759.2  Primer F 5' AGTTCAAGACCCAGATCC 3'  Primer R 5' TCGAAGACCGTAGAACAG 3' |
| Homo sapiens ubiquitin specific peptidase 3 (USP3), transcript variant 2, mRNA NM_001256702.1  Primer F 5' TTTAACTGTGCAGCCTTACC 3'  Primer R 5' GTGTTGCCACAAACTGTTTC 3' |
| Homo sapiens ubiquitin specific peptidase 4 (USP4), transcript variant 3, mRNA NM_001251877.2  Primer F 5' ACCGAGGCGTGGAATAAAC 3'  Primer R 5' TGGCAACTCAGCACATTGG 3' |
| Homo sapiens ubiquitin specific peptidase 5 (USP5), transcript variant 1, mRNA NM_001098536.2  Primer F 5' TTTGCCTCATTCCCTGACTACC 3'  Primer R 5' CGTCTTCGTTGCCATAGAAACC 3' |
| Homo sapiens ubiquitin specific peptidase 6 (USP6), transcript variant 1, mRNA NM_001304284.2  Primer F 5' CCTTGGGTGGGTGTAGGTATG 3'  Primer R 5' ATGCGAGGGATGGGAAGTG 3' |
| Homo sapiens ubiquitin specific peptidase 7 (USP7), transcript variant 2, mRNA NM_001286457.2  Primer F 5' GAGGCAACCTTTCAGTTC 3'  Primer R 5' CGTGGCATCACCATAATC 3' |
| Homo sapiens ubiquitin specific peptidase 8 (USP8), transcript variant 2, mRNA NM_001128610.3  Primer F 5' ATCATTCACCCACCAACAC 3'  Primer R 5' AGAAGCAGAAAGCCTTGAG 3' |
| Homo sapiens ubiquitin specific peptidase 9 X-linked (USP9X), transcript variant 3, mRNA NM_001039590.3  Primer F 5' CTGGCTGATGATTCCTCTG 3'  Primer R 5' TCTACACCTGGCTGACAAG 3' |
| **Homo sapiens ubiquitin specific peptidase 10 (USP10), transcript variant 1, mRNA**  NM_001272075.1  Primer F 5' GTGACACTTTGCCGAGAAC 3'  Primer R 5' TCCGCCTCCACATTAGAAC 3' |
| Homo sapiens ubiquitin specific peptidase 14 (USP14), transcript variant 2, mRNA NM_001037334.1  Primer F 5' GAAAGGAGGAACGCTAAAGG 3'  Primer R 5' CAGGCACAGAACGAATACAC 3' |
| **Homo sapiens ubiquitin specific peptidase 15 (USP15), transcript variant 1, mRNA**  NM_001252078.1  Primer F 5' TGCCTACTTCCAACTCTC 3'  Primer R 5' GCTCTTCCTTTCCTTCTC 3' |
| Homo sapiens ubiquitin specific peptidase 20 (USP20), transcript variant 2, mRNA NM_001008563.4  Primer F 5' GACCTTTGCCCTCACCTTG 3'  Primer R 5' CAGGCGTAACACCACAGTC 3' |
| Homo sapiens ubiquitin specific peptidase 22 (USP22), mRNA NM_015276.2  Primer F 5' GTGTAGTGTGCGGGACAGAG 3'  Primer R 5' AGGAGCGGGAGAGGGATAAG 3' |
| Homo sapiens ubiquitin specific peptidase 25 (USP25), transcript variant 1, mRNA NM_001283041.2  Primer F 5' GCCAAGCAGATACAAATG 3'  Primer R 5' TAGGTGTCCTCTTCAAAC 3' |
| Homo sapiens ubiquitin specific peptidase 28 (USP28), transcript variant 2, mRNA NM_001301029.2  Primer F 5' GCACAGAAGTTCGTTGTC 3'  Primer R 5' AAGTGGCAAGAGGCTTAG 3' |
| Homo sapiens ubiquitin specific peptidase 38 (USP38), transcript variant 2, mRNA NM_001290325.1  Primer F 5' TCTTTGGCGTCTTGCTTGTC 3'  Primer R 5' CGTATGCTTCCCTCTGTGTATG 3' |
| **Homo sapiens ubiquitin specific peptidase 39 (USP39), transcript variant 2, mRNA**  NM_001256725.1  Primer F 5' GCTGGACTTTGACTTTGAG 3'  Primer R 5' AATGTAGGCGTGAGACTTC 3' |
| Homo sapiens ubiquitin specific peptidase 47 (USP47), transcript variant 1, mRNA NM_001282659.2  Primer F 5' ATATGGCACCACTGGATCATAC 3'  Primer R 5' TCTTGGAAGCGGACCTATAAAC 3' |
| **Homo sapiens ubiquitin specific peptidase 49 (USP49), transcript variant 1, mRNA**  NM_001286554.1  Primer F 5' GAAGTGGTGCTGCTTAGAGTG 3'  Primer R 5' CCCGTCTCCTCAAAGTGTTTC 3' |
| Homo sapiens actin beta (ACTB), mRNA NM_001101.5  Primer F 5' GATGACCCAGATCATGTTTGAG 3'  Primer R 5' TAATGTCACGCACGATTTCC 3' |
| **Homo sapiens glutaminase (GLS), transcript variant 2, mRNA; nuclear gene for mitochondrial product**  NM_001256310.2  Primer F 5' CTGTGCTCCATTGAAGTG 3'  Primer R 5' TGCCCTGAGAAGTCATAC 3' |
| **hsa-miR-146a-5p MIMAT0000449** Primer RT  5' GTCGTATCCAGTGCAGGGTCCGAGGTATTCGCACTGGATACGACAACCCA 3'  Primer F 5' CGCGTGAGAACTGAATTCCA 3'  Primer R 5' AGTGCAGGGTCCGAGGTATT 3' |
| Homo sapiens RNA, U6 small nuclear 1 (RNU6-1), small nuclear RNA NR_004394.1  Primer F 5' CTCGCTTCGGCAGCACA 3'  Primer R 5' AACGCTTCACGAATTTGCGT 3' |

**Table S2.** Antibodies for Western bot.

| Antibody | Company | Catalog number | Dilution factor |
| --- | --- | --- | --- |
| USP6 | Abcam | Ab224725 | 1:500 |
| GLS1 | Abcam | Ab156876 | 1:3000 |
| CD81 | Abcam | Ab109201 | 1:700 |
| TSG101 | Abcam | Ab125011 | 1:1000 |
| Alix | Abcam | Ab225555 | 1:3000 |
| Ubiquitin | Abcam | Ab134953 | 1:1000 |
| β-actin | Cell Signaling Technology | #4970 | 1:1000 |

**Table S3.** Target sequences of human USP6 and GLS1 shRNAs.

| **shRNA** | **Target sequence** |
| --- | --- |
| shUSP6-1 | 5’ GGCTGTTTCTTCAGCATGA 3’ |
| shUSP6-2 | 5’ GGTGGTTCAAGGTGAATAA 3’ |
| shUSP6-3 | 5’ CCGTTAGATTCTCACAGAA 3’ |
| shGLS1-1 | 5’ GCTCTCCTTCGGAGATCTT 3’ |
| shGLS1-2 | 5’ CCACATAATCCTATGGTAA 3’ |
| shGLS1-3 | 5’ GGTGGTGATCAAAGGGTAA 3’ |

**Table S4.** List of proteins identified by mass spectrometry.

| **Protein IDs** | **Protein names** | **Gene names** | **Sequence length** | **Peptides** | **Unique peptides** | **Sequence coverage [%]** | **Unique sequence coverage [%]** | **Mol. weight [kDa]** | **Q-value** | **Score** | **Intensity** | **MS/MS Count** |
| --- | --- | --- | --- | --- | --- | --- | --- | --- | --- | --- | --- | --- |
| P35125 | Ubiquitin Specific Peptidase 6 | USP6 | 376 | 3 | 3 | 12.2 | 12.2 | 158.658 | 0 | 30.552 | 59464000 | 3 |
| O00231 | 26S proteasome non-ATPase regulatory subunit 11 | PSMD11 | 422 | 12 | 12 | 37 | 37 | 47.463 | 0 | 185.55 | 3035300000 | 11 |
| O00303 | Eukaryotic translation initiation factor 3 subunit F | EIF3F | 357 | 4 | 4 | 18.8 | 18.8 | 37.563 | 0 | 81.47 | 1731100000 | 4 |
| O00410 | Importin-5 | IPO5 | 1097 | 2 | 2 | 1.4 | 1.4 | 123.63 | 0 | 28.336 | 29433000 | 2 |
| **O94925** | **Glutaminase 1** | **GLS1** | **375** | **5** | **5** | **22.9** | **22.9** | **73.461** | **0** | **87.602** | **869830000** | **6** |
| O00442 | RNA 3-terminal phosphate cyclase | RTCA | 366 | 3 | 3 | 8.2 | 8.2 | 39.336 | 0 | 29.734 | 188200000 | 1 |
| O14773 | Tripeptidyl-peptidase 1 | TPP1 | 563 | 2 | 2 | 5 | 5 | 61.247 | 0 | 38.833 | 263530000 | 2 |
| O14929 | Histone acetyltransferase type B catalytic subunit | HAT1 | 419 | 4 | 4 | 13.4 | 13.4 | 49.512 | 0 | 110.84 | 2430800000 | 11 |
| O15160 | DNA-directed RNA polymerases I and III subunit RPAC1 | POLR1C | 346 | 6 | 6 | 27.2 | 27.2 | 39.249 | 0 | 104.71 | 1311200000 | 6 |
| O15264 | Mitogen-activated protein kinase 13 | MAPK13 | 365 | 2 | 2 | 6.8 | 6.8 | 42.089 | 0 | 15.312 | 89275000 | 3 |
| O15372 | Eukaryotic translation initiation factor 3 subunit H | EIF3H | 352 | 3 | 3 | 14.8 | 14.8 | 39.93 | 0 | 44.418 | 388870000 | 3 |
| O43615 | Mitochondrial import inner membrane translocase subunit TIM44 | TIMM44 | 452 | 7 | 7 | 20.6 | 20.6 | 51.355 | 0 | 109.91 | 859610000 | 9 |
| O43837 | Isocitrate dehydrogenase [NAD] subunit beta, mitochondrial | IDH3B | 385 | 2 | 2 | 8.1 | 8.1 | 42.183 | 0 | 34.196 | 171750000 | 2 |
| O43852 | Calumenin | CALU | 315 | 8 | 8 | 32.1 | 32.1 | 37.106 | 0 | 201.17 | 2578200000 | 15 |
| O43929;CON__Q2YDI2 | Origin recognition complex subunit 4 | ORC4 | 436 | 3 | 3 | 9.6 | 9.6 | 50.377 | 0 | 43.106 | 114680000 | 3 |
| O60256 | Phosphoribosyl pyrophosphate synthase-associated protein 2 | PRPSAP2 | 369 | 2 | 1 | 9.8 | 6 | 40.925 | 0 | 7.5619 | 43735000 | 1 |
| O60547 | GDP-mannose 4,6 dehydratase | GMDS | 372 | 4 | 4 | 17.7 | 17.7 | 41.949 | 0 | 51.619 | 616180000 | 4 |
| O60664 | Perilipin-3 | PLIN3 | 434 | 13 | 13 | 33.9 | 33.9 | 47.074 | 0 | 323.31 | 6305600000 | 19 |
| O60884 | DnaJ homolog subfamily A member 2 | DNAJA2 | 412 | 5 | 5 | 17.5 | 17.5 | 45.745 | 0 | 69.009 | 2230100000 | 5 |
| O75306 | NADH dehydrogenase [ubiquinone] iron-sulfur protein 2, mitochondrial | NDUFS2 | 463 | 4 | 4 | 17.7 | 17.7 | 52.545 | 0 | 69.824 | 593640000 | 5 |
| O75390 | Citrate synthase, mitochondrial | CS | 466 | 6 | 6 | 28.8 | 28.8 | 51.712 | 0 | 98.786 | 1929000000 | 5 |
| O75439 | Mitochondrial-processing peptidase subunit beta | PMPCB | 489 | 2 | 2 | 8 | 8 | 54.366 | 0 | 24.368 | 512700000 | 1 |
| O75689 | Arf-GAP with dual PH domain-containing protein 1 | ADAP1 | 374 | 4 | 4 | 16.3 | 16.3 | 43.395 | 0 | 32.398 | 212170000 | 4 |
| O75821 | Eukaryotic translation initiation factor 3 subunit G | EIF3G | 320 | 7 | 7 | 23.4 | 23.4 | 35.611 | 0 | 153.99 | 4215300000 | 10 |
| O75844 | CAAX prenyl protease 1 homolog | ZMPSTE24 | 475 | 3 | 3 | 9.7 | 9.7 | 54.812 | 0 | 37.775 | 263690000 | 2 |
| O75874 | Isocitrate dehydrogenase [NADP] cytoplasmic | IDH1 | 414 | 10 | 10 | 33.8 | 33.8 | 46.659 | 0 | 170.28 | 5156000000 | 18 |
| O75955 | Flotillin-1 | FLOT1 | 427 | 6 | 6 | 19.7 | 19.7 | 47.355 | 0 | 91.402 | 610830000 | 6 |
| O76003 | Glutaredoxin-3 | GLRX3 | 335 | 3 | 3 | 20.6 | 20.6 | 37.432 | 0 | 67.856 | 253890000 | 3 |
| O94766;REV__Q9Y2Y4;REV__Q9BRK4 | Galactosylgalactosylxylosylprotein 3-beta-glucuronosyltransferase 3 | B3GAT3 | 335 | 3 | 3 | 15.5 | 15.5 | 37.121 | 0 | 48.282 | 97960000 | 3 |
| O94905;O75477 | Erlin-2 | ERLIN2 | 339 | 5 | 5 | 19.2 | 19.2 | 37.839 | 0 | 49.777 | 750430000 | 4 |
| O95433 | Activator of 90 kDa heat shock protein ATPase homolog 1 | AHSA1 | 338 | 4 | 4 | 19.5 | 19.5 | 38.274 | 0 | 99.111 | 3589300000 | 5 |
| O95758 | Polypyrimidine tract-binding protein 3 | PTBP3 | 552 | 4 | 4 | 12.1 | 12.1 | 59.689 | 0 | 54.208 | 400880000 | 5 |
| O95825 | Quinone oxidoreductase-like protein 1 | CRYZL1 | 349 | 2 | 2 | 7.7 | 7.7 | 38.696 | 0 | 21.439 | 61252000 | 2 |
| O96019 | Actin-like protein 6A | ACTL6A | 429 | 2 | 2 | 10.3 | 10.3 | 47.46 | 0 | 35.128 | 379930000 | 2 |
| P00505 | Aspartate aminotransferase, mitochondrial | GOT2 | 430 | 4 | 4 | 15.1 | 15.1 | 47.517 | 0 | 69.548 | 2200600000 | 5 |
| P00558;P07205 | Phosphoglycerate kinase 1 | PGK1 | 417 | 14 | 14 | 42 | 42 | 44.614 | 0 | 253.01 | 23468000000 | 33 |
| P00966 | Argininosuccinate synthase | ASS1 | 412 | 8 | 8 | 34 | 34 | 46.53 | 0 | 124.95 | 5514600000 | 10 |
| P01892;P10316;P01891;P30512;P30459;P30457;P30456;P30453;P30450;P18462;P16190;P16189;P10314 | HLA class I histocompatibility antigen, A-2 alpha chain;HLA class I histocompatibility antigen, A-69 alpha chain | HLA-A | 365 | 7 | 4 | 32.1 | 16.4 | 40.921 | 0 | 46.058 | 210600000 | 5 |

**Table S5.** Antibodies for flow cytometry analysis.

| Antibody | Company | Catalog number |
| --- | --- | --- |
| CD90 Monoclonal Antibody , FITC | eBioscience™ | 11-0909-42 |
| CD44 Monoclonal Antibody , FITC | eBioscience™ | 11-0441-82 |
| CD105 Monoclonal Antibody , PE | eBioscience™ | 12-1057-42 |
| CD11b Monoclonal Antibody , PE | eBioscience™ | 12-0118-42 |
| CD34 Monoclonal Antibody , PE | eBioscience™ | 12-0349-42 |
| CD45 Monoclonal Antibody , FITC | eBioscience™ | 11-0459-42 |

**Supplemental Materials**

**IP and LC/MS analysis**

Proteins were extracted from K562-R cells, pre-cleared with protein A/G beads, and incubated with anti-USP6 (Bethylaboratories, A305-225) or control IgG for 12 h. The immunoprecipitated protein complexes were separated by SDS-PAGE, and stained with Coomassie Brilliant Blue. Several differently migrating bands were excised, digested, and analyzed by LC/MS following the reported protocol (Tang et al., 2013).

**Isolation and culture of hucMSC**

HucMSCs were isolated according to the Laboratory of Stem Cell, Institute of Tropical Disease, Airlangga University’s protocol. Cells were maintained in collagen-coated dishes using α-MEM with FBS and leukemia inhibitory factor (Gibco, Gaithersburg, MD). CD63, CD9 and Alix were measured by immublots. The immunophenotype of hucMSC was characterized using flow cytometry (FACSCalibur; BD Biosciences, San Jose, CA). Antibodies for flow cytometry analysis were shown in Table S5.

**Exosomes isolation**

Exosomes were isolated using VEX Exosome Isolation Reagent (Beyotime). Final exosomes were resuspended in PBS and stored at −70°C. The morphology was observed using transmission electron microscopy (FEI Tecnai 12, Philips, The Netherlands). The Alix, TSG101 and CD81 molecules were analyzed using Western blot.


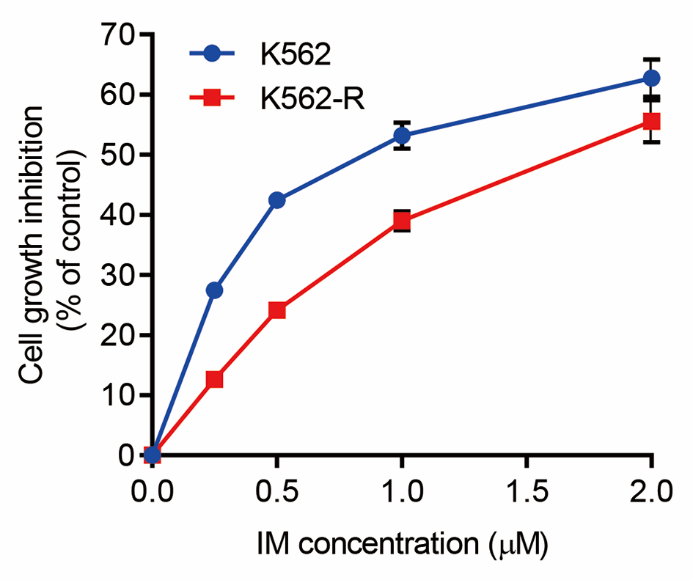


**Figure S1.** The IC50 (half-maximal inhibitory concentration) of IM in K562 and K562-R cells. K562 and K562-R cells in 96-well plates were incubated with IM at an increased concentration (0, 0.25, 0.5, 1.0 and 2.0 μg/ml) for 48 h. Cell Counting Kit-8 (CCK-8) assay was performed to determined cell growth inhibition ratio.


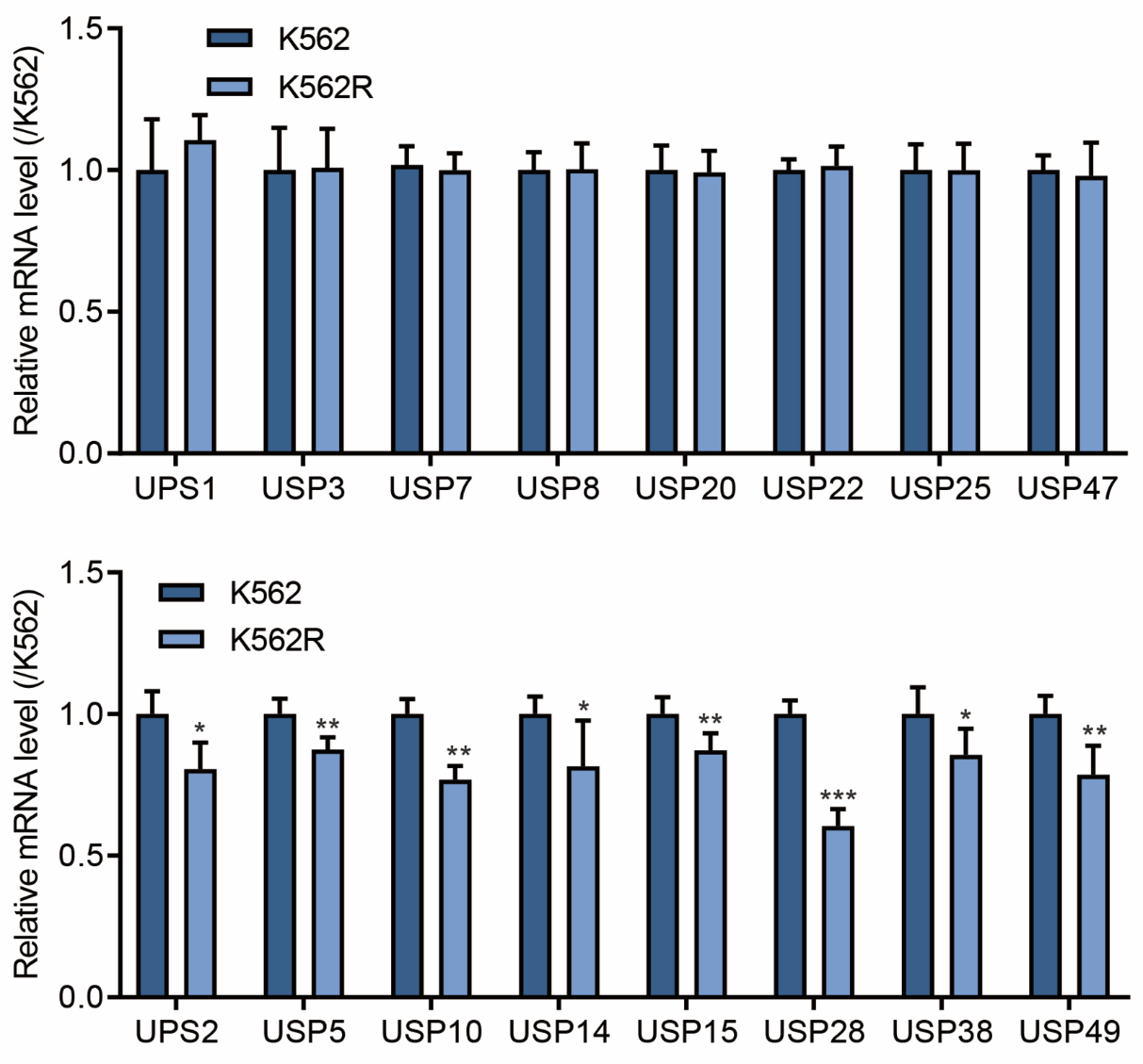


**Figure S2.** The mRNA levels of USP family members in K562-R and K562 cells were detected by qPCR. *P<0.05, **P<0.01, ***P<0.001 vs K562.


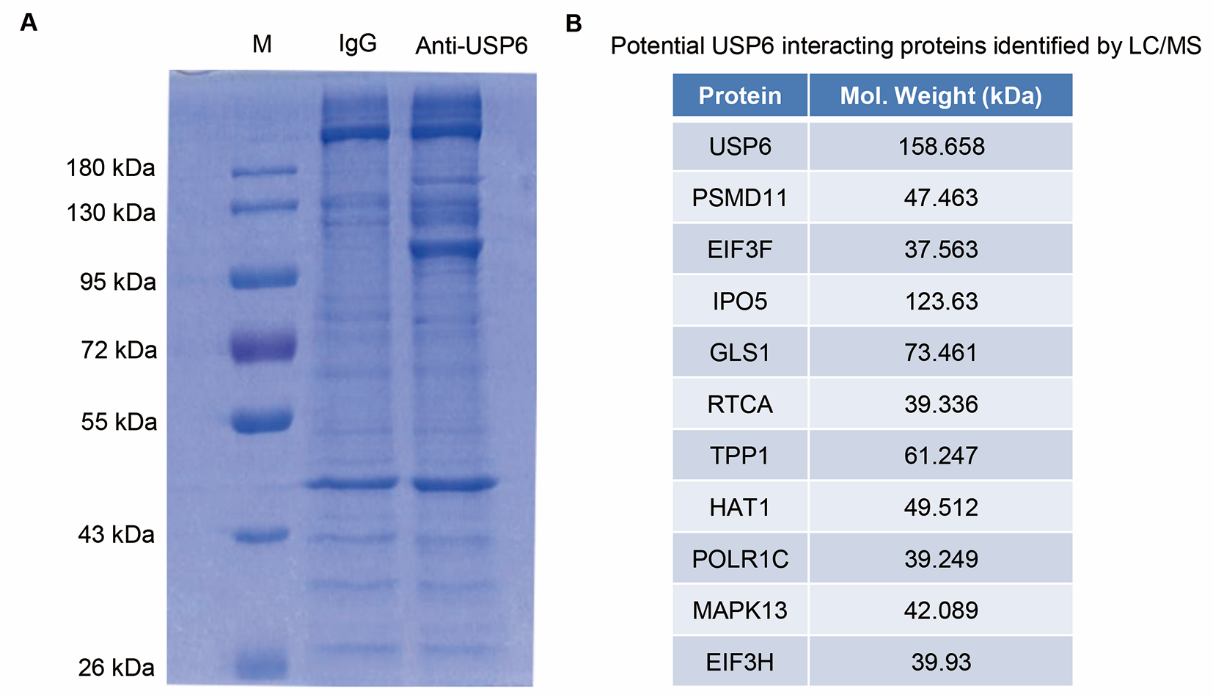


**Figure S3.** Identifying USP6 binding protein by IP and mass spectrometry. (A) Purification of the USP6 complex was carried out according to the procedure described in Materials and Methods. Proteins were separated on SDS-PAGE and stained with Coomassie Blue. (B) List of USP6-associated proteins identified by mass spectrometry analysis.


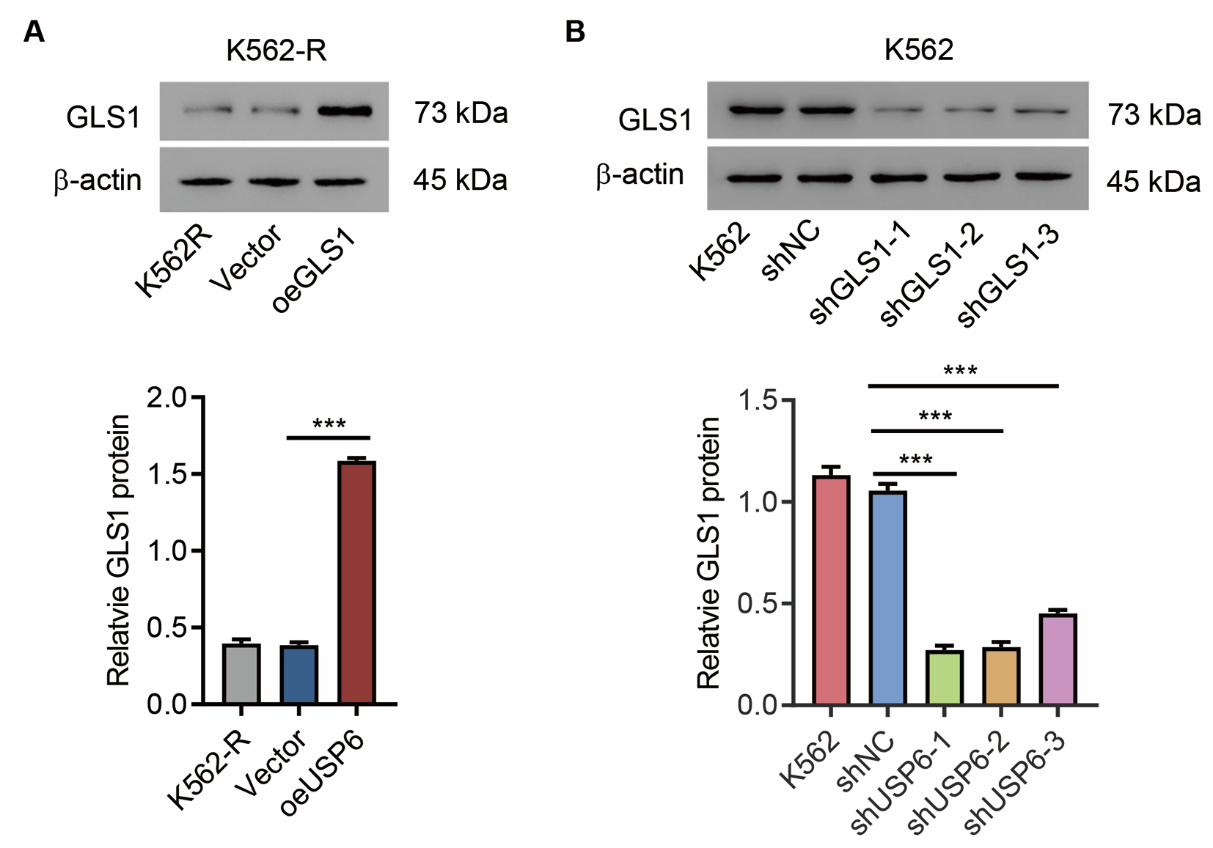


**Figure S4.** GLS1 expression in K562-R and K562 cells. Western blot analysis of GLS1 expression in (A) K562-R cells infected with GLS1 gene overexpression lentivirus (oeGLS) or control lentivirus (Vector), and (B) K562 cells infected with GLS1 gene interference lentivirus (shGLS-1, -2, -3) or control (shNC) lentivirus. ***P<0.001.


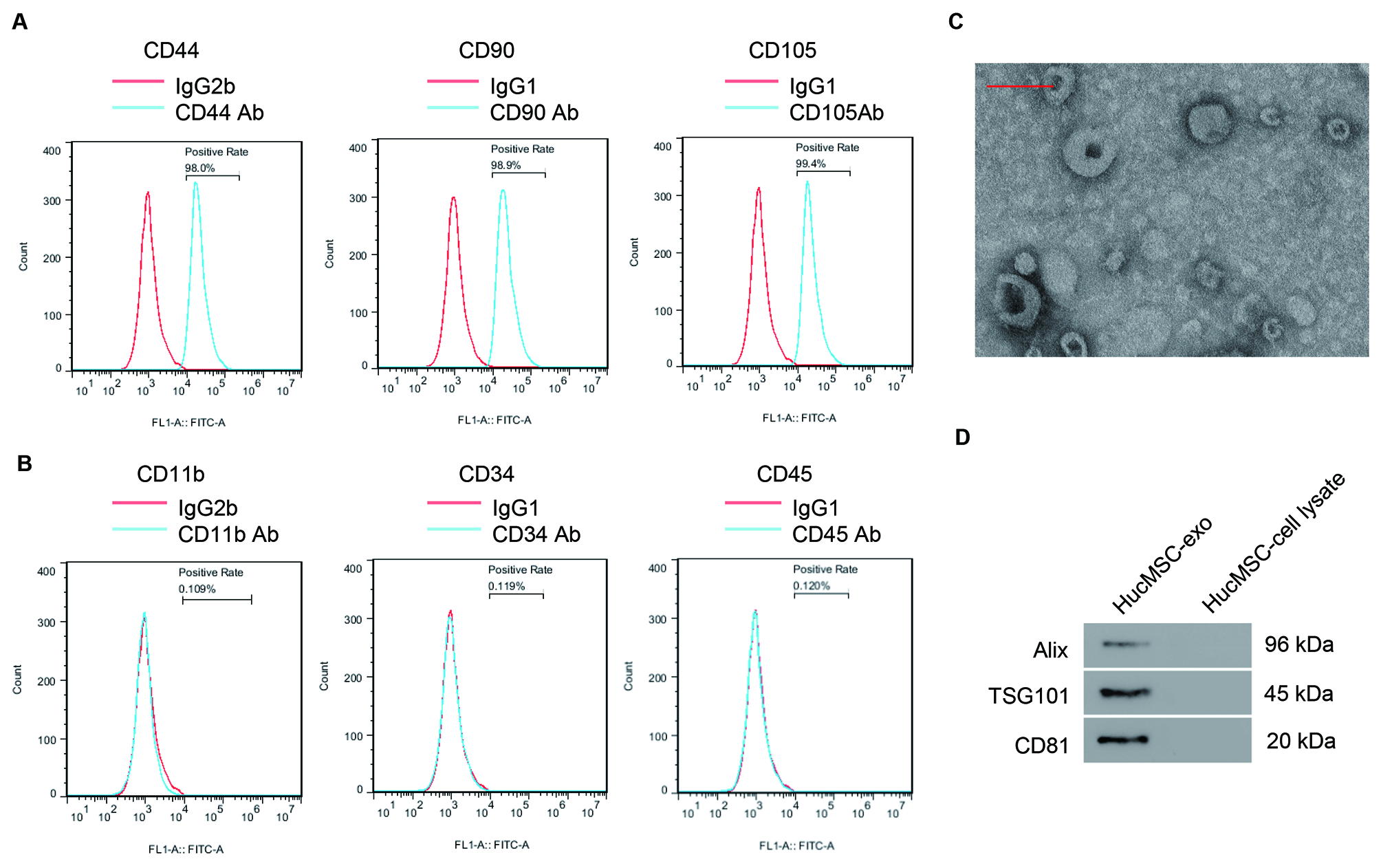


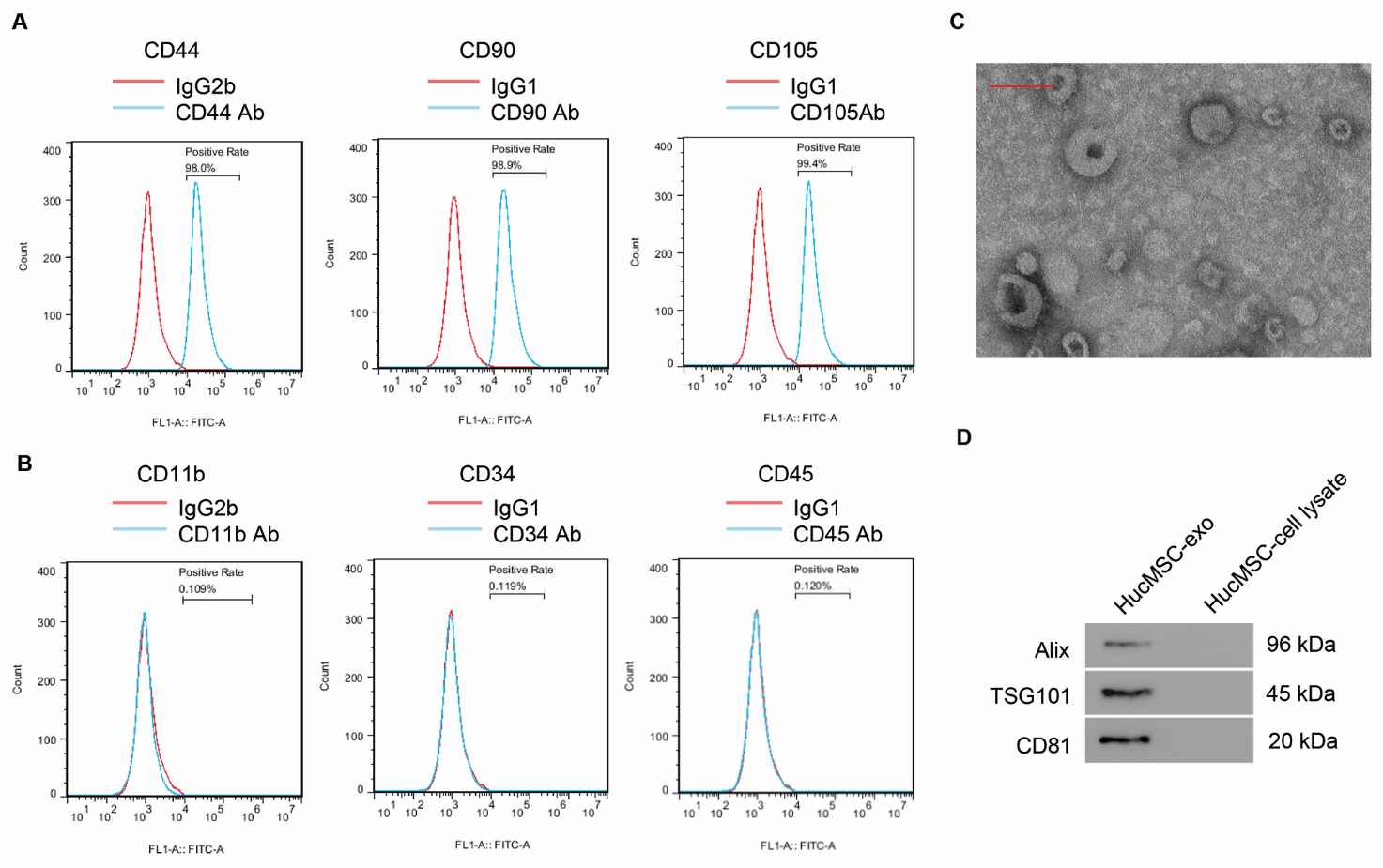


**Figure S5.** Isolating hucMSC-exo. (A-B) HucMSC surface markers analysis. (C) Morphology analysis. Scale bar: 100 nm. (D) Western blot analysis of Alix, TSG101 and CD81.


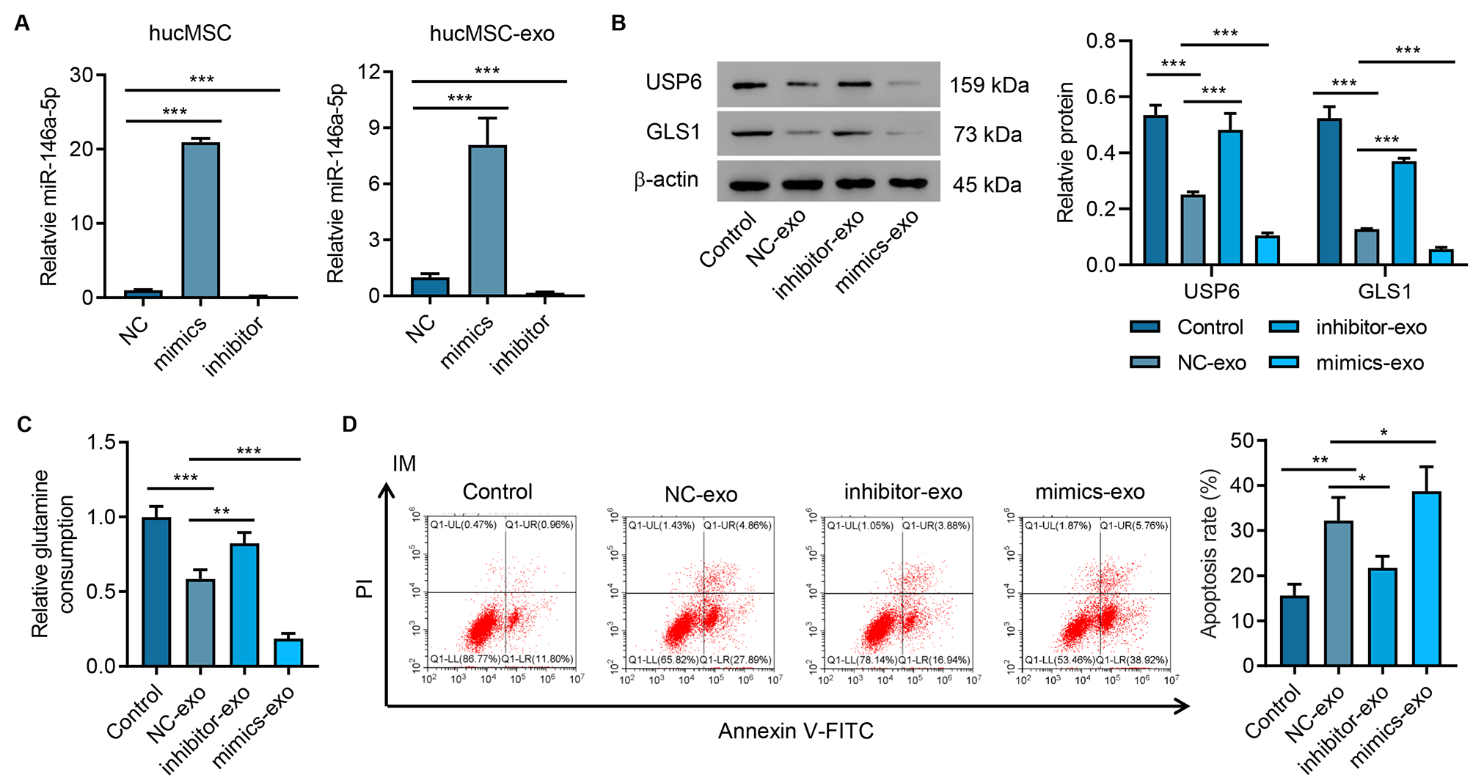


**Figure S6.** HucMSC exosome promoted IM-induced apoptosis in K562-R cells through miR-145a-5p. Transfection of miR-146a-5p mimics, inhibitor or control (NC). (A) miR-146a-5p levels in cells (left) or exosomes (right) were detected. K562-R cells were treated with the hucMSC-exo mentioned before. (B) The expression of USP6 and GLS1 were detected. (C) Biochemical detection of glutamine uptake. (D) IM-induced apoptosis. *P<0.05, **P<0.01, ***P<0.001.
