# Supplementary material for: HucMSC exosomes promoted imatinib-induced apoptosis in K562-R cells via a miR-145a-5p/USP6/GLS1 axis
Source: Cell Death Dis. 2022 Jan 28;13(1):92. doi: 10.1038/s41419-022-04531-3 (PMC8799639; doi:10.1038/s41419-022-04531-3)
Supplement: Supplementary file 3 — CERTIFICATE OF EDITING [file 41419_2022_4531_MOESM3_ESM.pdf]

enago
Author First, Quality First
www.enago.cn

CERTIFICATE OF EDITING

This is to certify that the paper titled **HucMSC exosomes promoted imatinib-induced apoptosis in K562-R cells via a miR-145a-5p/USP6/GLS1 axis** has been edited for English language, grammar, punctuation, and spelling by Enago, the editing brand of Crimson Interactive Consulting Co. Ltd.

ISO 17100:2015
Translation Service Providers

ISO 27001:2013
Information Security Management System

ISO 9001:2015
Quality Management System

Issued by:
Enago, Crimson Interactive (Beijing) Consulting Co., Ltd.
Room 607, Zhucheng Building,
No. 6 Zhongguancun South Street,
Haidian District, Beijing

Disclaimer: The intent of the author's message has been preserved during the editing process. The author is free to accept or reject our changes in the document after reviewing our edits. This certificate has been awarded at the time of sharing the final edited version (full file or sections of the file) with the author. Enago does not bear any responsibility for any alterations done by the author to the edited document post 19th November 2021.

Japan	www.enago.jp, www.ulatus.jp, www.voxtab.jp	Russia	www.enago.ru
Taiwan	www.enago.tw, www.ulatus.tw	Arabic	www.enago.ae
China	www.enago.cn, www.ulatus.cn	Turkey	www.enago.com.tr
Brazil	www.enago.com.br, www.ulatus.com.br	S. Korea	www.enago.co.kr
Germany	www.enago.de	Global	www.enago.com, www.ulatus.com, www.voxtab.com

About Crimson:
Crimson Interactive Consulting Co. Ltd. is one of the world's leading academic research support services. Since 2005, we've supported over 2 million researchers in 125 countries with their publication goals.
