# Supplementary material for: HucMSC exosomes promoted imatinib-induced apoptosis in K562-R cells via a miR-145a-5p/USP6/GLS1 axis
Source: Cell Death Dis. 2022 Jan 28;13(1):92. doi: 10.1038/s41419-022-04531-3 (PMC8799639; doi:10.1038/s41419-022-04531-3)

I and other authors agree to add “Yixin Chen” as the author of the paper "HucMSC exosomes promoted imatinib-induced apoptosis in K562-R cells via a miR-145a-5p/USP6/GLS1 axis"

All of the co-authors’ email responses are list as below:

Xiaowen Chen ([cxwbrian@aliyun.com](mailto:cxwbrian@aliyun.com)) response


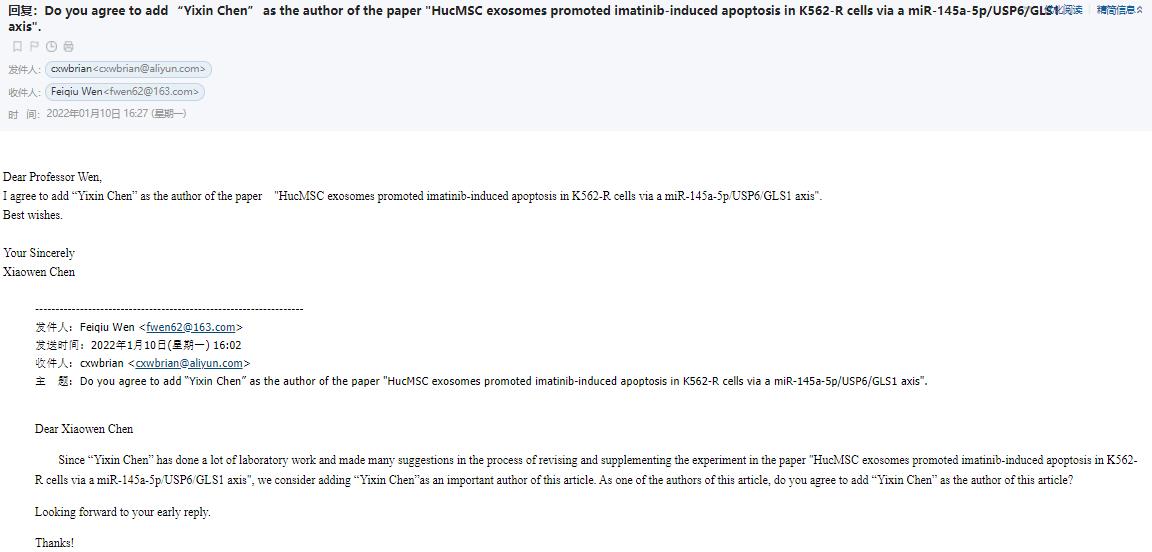


Yixin Chen ([yixinchen2013@126.com](mailto:yixinchen2013@126.com)) response


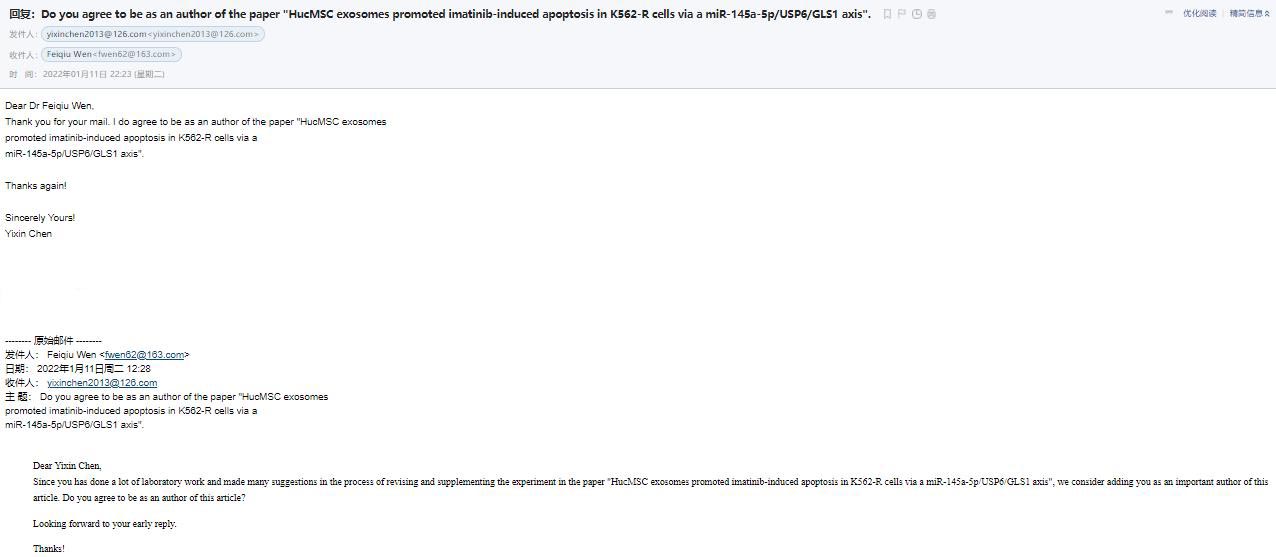


Min Zhang ([zhangmin832@163.com](mailto:zhangmin832@163.com)) response


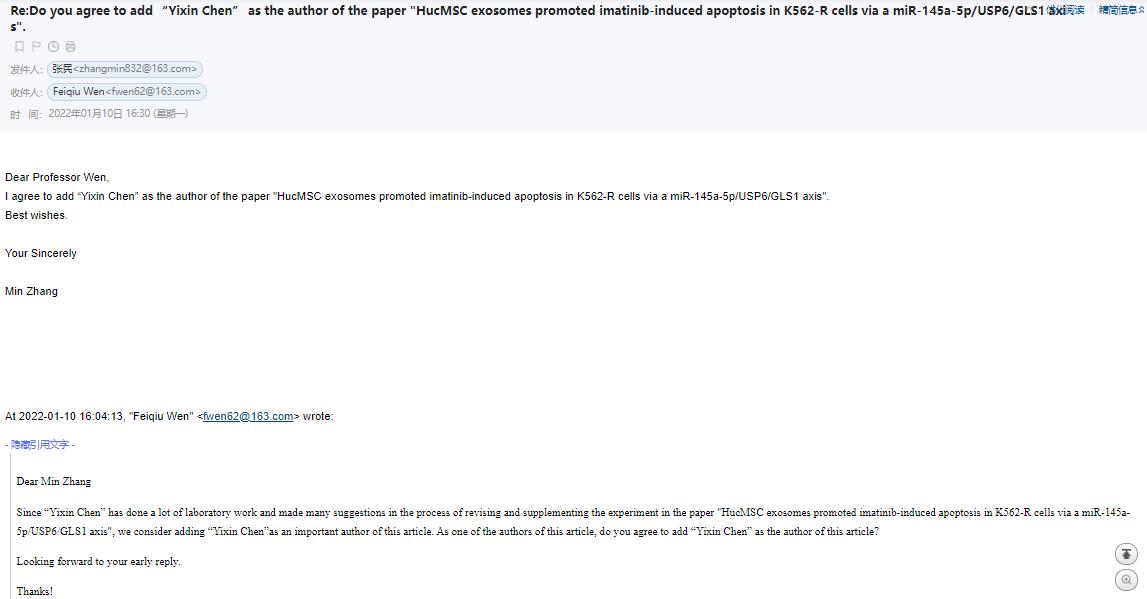


Hui Cheng ([chenghui19831103@163.com](mailto:chenghui19831103@163.com)) response


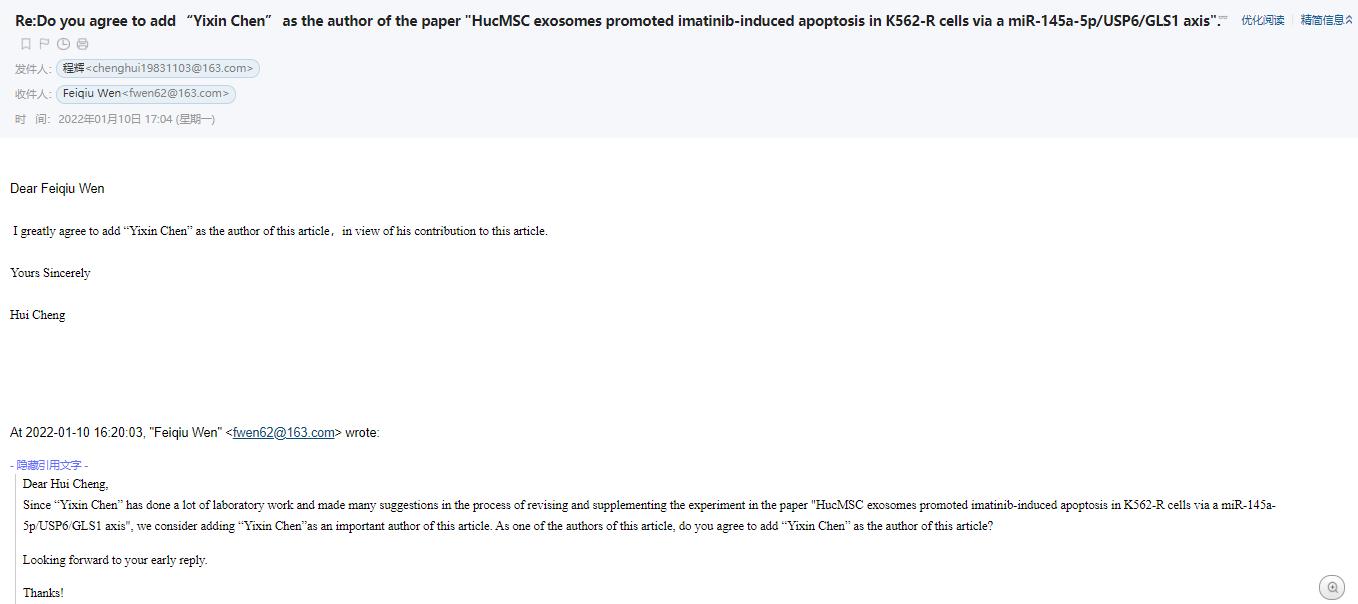


Huirong Mai ([maihuirong@163.com](mailto:maihuirong@163.com)) response


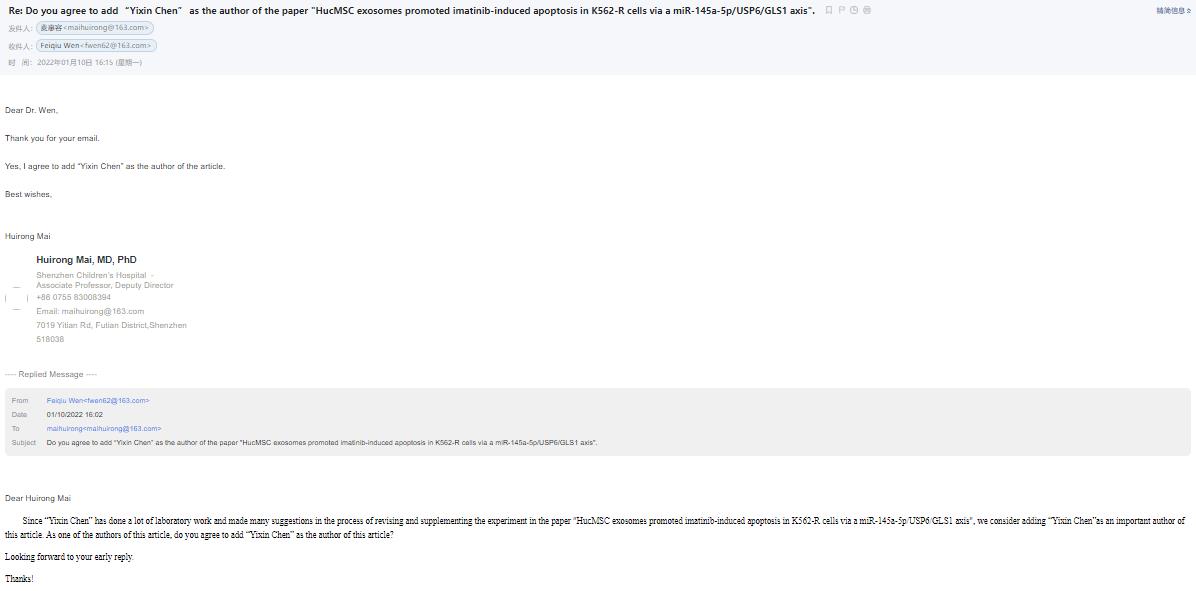


Meng Yi ([doctor_eva@163.com](mailto:doctor_eva@163.com)) response


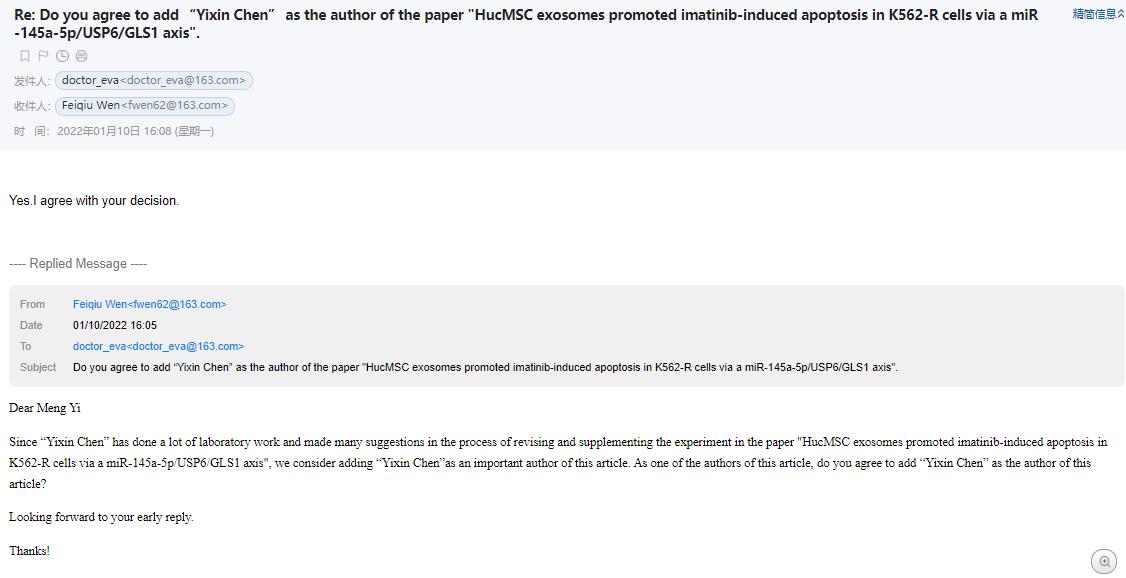


Huanli Xu ([xuhuanli35@163.com](mailto:xuhuanli35@163.com)) response


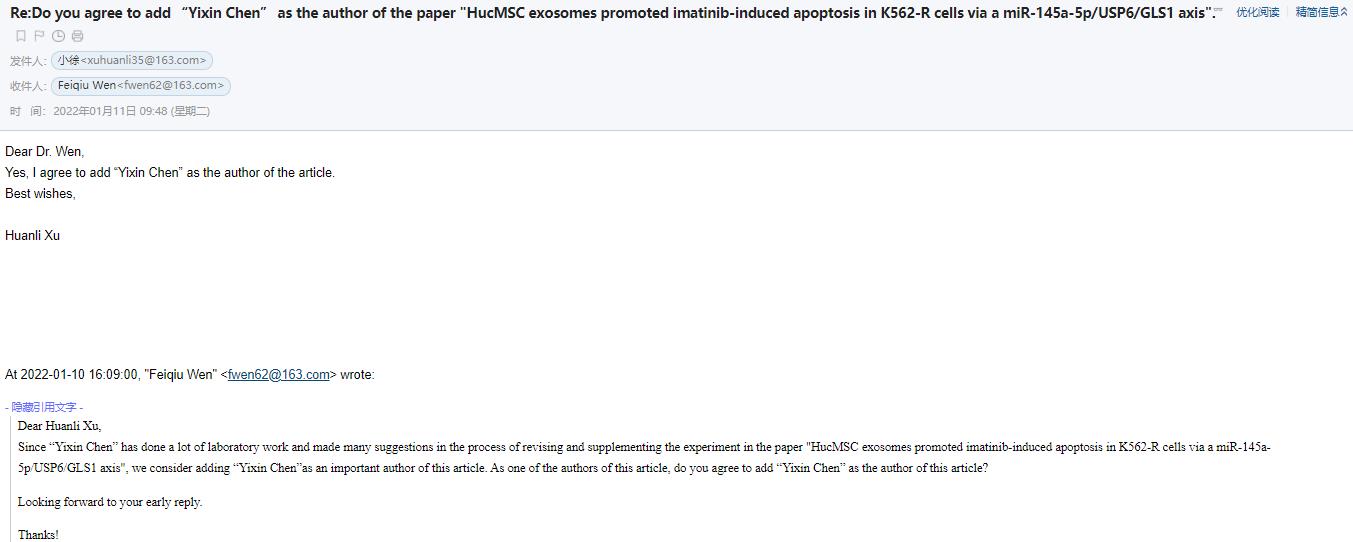


Xiuli Yuan ([yuanxiuli021@sohu.com](mailto:yuanxiuli021@sohu.com)) response


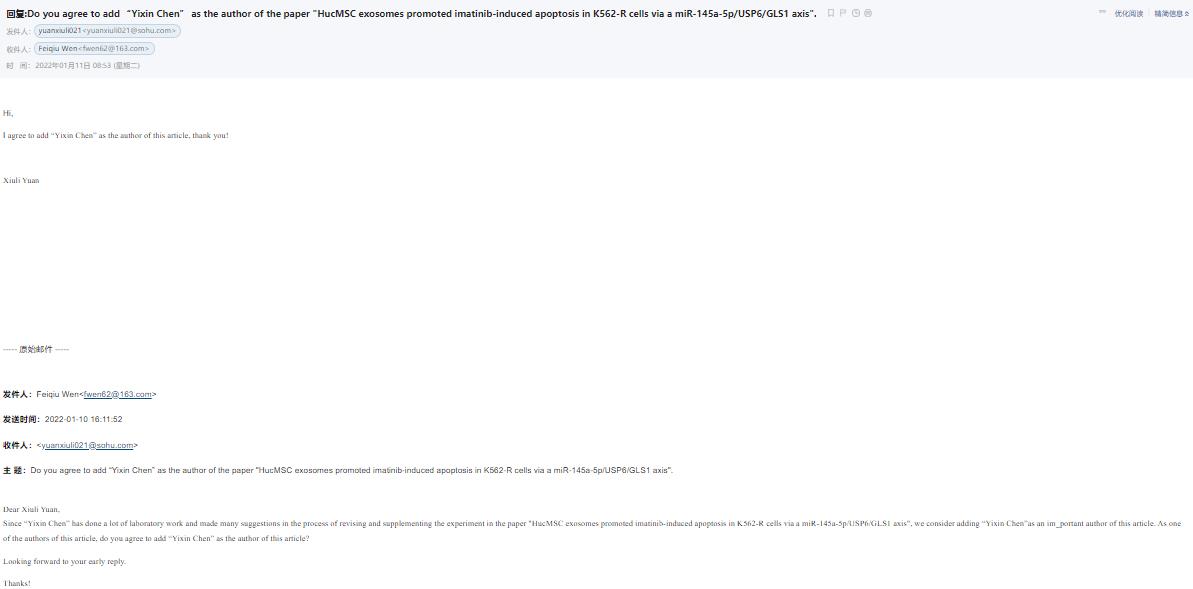


Sixi Liu ([tiger647@sina.com](mailto:tiger647@sina.com)) response


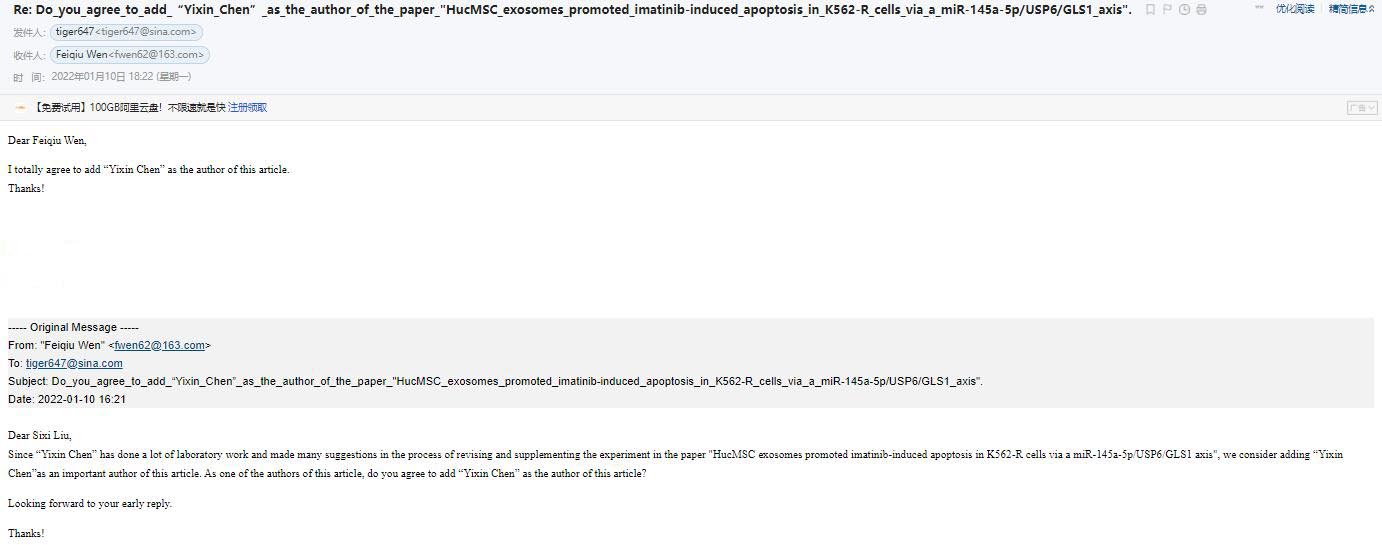

Supplement: Supplementary file 4 — co-authors’ email responses [file 41419_2022_4531_MOESM4_ESM.docx]
